# Supplementary material for: Length-dependent motions of SARS-CoV-2 frameshifting RNA pseudoknot and alternative conformations suggest avenues for frameshifting suppression
Source: Nat Commun. 2022 Jul 25;13:4284. doi: 10.1038/s41467-022-31353-w (PMC9310368; doi:10.1038/s41467-022-31353-w)
Supplement: Supplementary file 1 — Supplementary Information [file 41467_2022_31353_MOESM1_ESM.pdf]

Supplementary Information for Length-dependent motions of SARS-CoV-2 frameshifting RNA pseudoknot and alternative conformations suggest avenues for frameshifting suppression

Shuting Yan,[†,‡] Qiyao Zhu,[†,¶] Swati Jain,[‡] and Tamar Schlick[*,‡,¶,§]

†*These authors contributed equally to this work.*

‡*Department of Chemistry, New York University, New York, NY 10003 U.S.A.*

¶*Courant Institute of Mathematical Sciences, New York University, New York, NY 10012 U.S.A.*

§*NYU-ECNU Center for Computational Chemistry, NYU Shanghai, Shanghai 200062, P.R. China*

E-mail: schlick@nyu.edu

1 FSE mutation maps

We produce mutation maps for the 84-nt FSE region and the 3822-nt spike gene region of the five major COVID variants (Alpha, Beta, Gamma, Delta, and Omicron) (Supplementary Fig. 1). 3575 available Alpha and 898 Beta variant RNA sequences are downloaded from GISAID[1] on February 8, 2021; 1170 Gamma and 1000 Delta sequences are randomly selected from those downloaded on July 8, 2021; 182 available Omicron sequences are downloaded on November 30, 2021. We then align them with the official SARS-CoV-2 RNA reference sequence of 29891-nt provided by GISAID (Accession ID: EPI_ISL_402124), following the same protocol used in our prior paper[2]. The FSE region occupies residues 13462–13545, and the spike gene region occupies 21564–25384.

The FSE region mostly has a single-nucleotide mutation in $<1\%$ variant sequences (Supplementary Fig. 1). For the Alpha variant, only 4 out of 3575 sequences have point mutations (C13506U twice, C13517U once, and A13533G once); for Beta and Gamma, only 5/898 and 4/1170, and besides the common C13517U mutation, we observe some new mutations (A13482G, A13512G, and U13527C for Beta, C13501U and C13536 for Gamma); for Delta and Omicron, only 2/1000 and 1/182, and no new mutations.

We label all the FSE mutations in the three conformations 3_6, 3_3, and 3_5 (Supplementary Fig. 1). These mutations are either in Stems 1 and 3, or in the loop regions. They are all transition mutations, i.e., pyrimidine-pyrimidine or purine-purine, and as a result, the 2D structures would not be affected. For example, the A13482G mutation appeared in Beta, Delta, and Omicron variants locates in the $5'$ strand of Stem 1, forming an A-U base pair in the wildtype and a G-U wobble base pair in the mutant.

On the other hand, the spike gene can have dozens of mutations per sequence, and new mutations are added every time when a new variant appears. All Alpha sequences have 5-11 mutations in the spike gene, and there are 7 high frequency mutations that occur in $>70\%$ of the sequences; for Beta, 4-12 mutations per sequence, and 7 high frequency mutations; for Gamma, 2-25 mutations per sequence, and 12 high frequency mutations. Noticeably, these high frequency mutations are mostly different for each variant. More mutations emerge since the Delta variant, having 8-24 mutations per sequence, and 18 high frequency mutations. For Omicron, 15-45 mutations are possible per sequence, and out of 36 high frequency mutations, only 5 have been seen in the previous variants.

Therefore, while the spike gene region is constantly subject to new mutations that change the translated protein structures, the FSE region has very few mutations, even for the Delta and Omicron variants. Moreover, the FSE mutations stay in the same set for all the variants, without introducing new ones, and they seem to maintain the FSE conformation by forming alternative Wobble base pairs or mutating the loop regions. This high conservation thus makes FSE a good drug target.

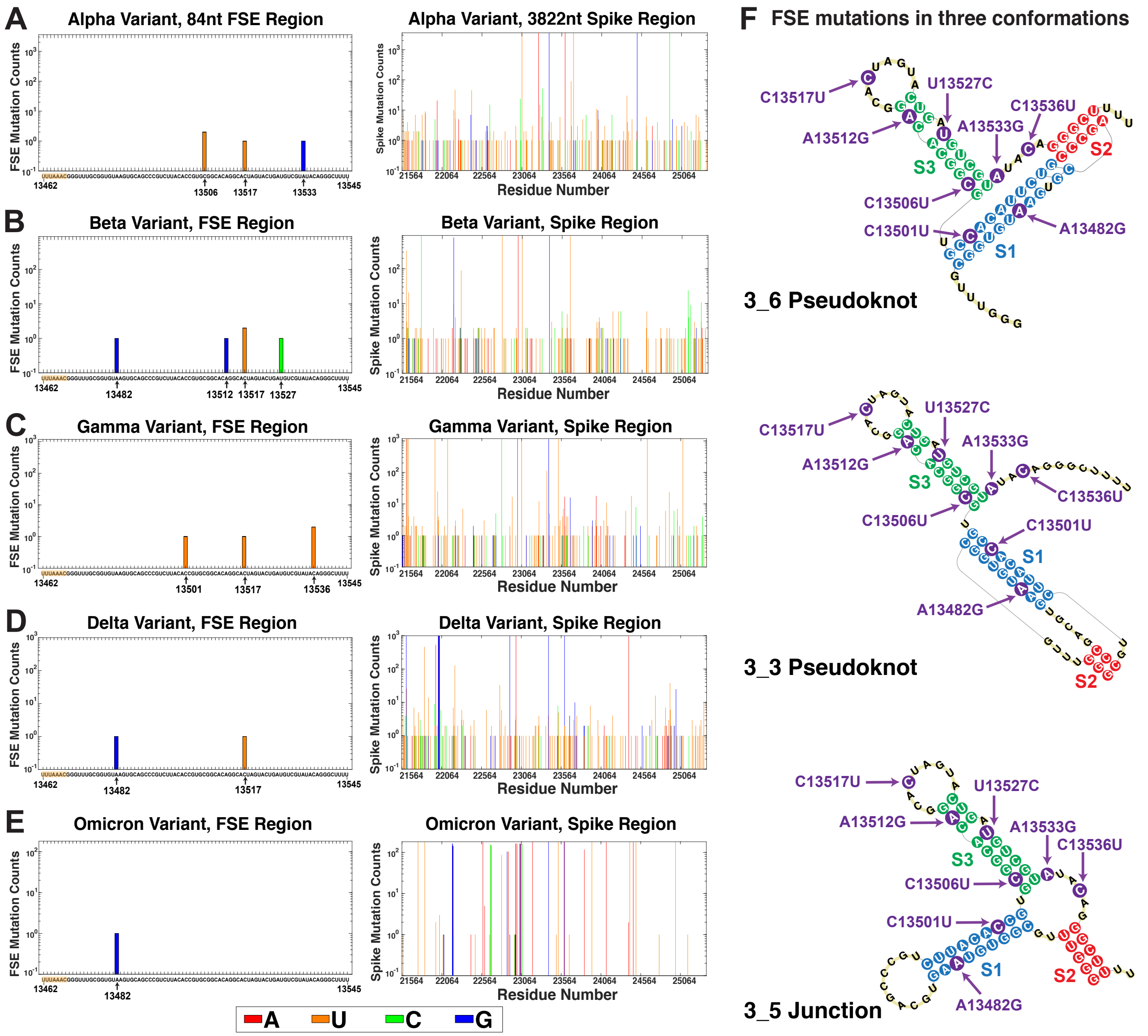

Supplementary Figure 1: SARS-CoV-2 RNA mutation maps for the (A) Alpha variant, (B) Beta variant, (C) Gamma variant, (D) Delta variant, and (E) Omicron variant for the 84-nt FSE region and the spike gene region. (F) The mutations in the FSE region are labeled in the three motifs 3_6, 3_3, and 3_5.

2 Wildtype FSE model validation

2.1 Initial 3D model validation

There are 26 initial 3D models: 12 predictions by four programs RNAComposer[3], SimRNA[4], iFoldRNA[5], and Vfold3D[6] for the three motifs 3_6, 3_3, and 3_5 at 77-nt; 8 predictions by the same four programs for the two pseudoknots 3_6 and 3_3 at 87-nt (3_5 not modeled at this length as it is only observed in the 77-nt landscape); 6 predictions by three programs RNAComposer, iFoldRNA, and Farfar2[7] for the two pseudoknots at 144-nt (SimRNA and Vfold3D failed to produce models at this length).

For each model, we extract the 2D structure using DSSR[8] and describe it in the dot-bracket notation: '.' for single nucleotides, '( ) [ ]' for base pairs. We then check whether the desired motif (3_6, 3_3, or 3_5) was generated. In addition, we calculate the Hamming distance between the SHAPE-directed and the model's 2D structure, i.e., the number of positions where nucleotides have different dot-bracket symbols (see Supplementary Table 1). If the predicted model yields the desired motif and has a Hamming distance ≤ 10, it is accepted (highlighted in green); otherwise, we reject it (red).

Of the 26 models above, we exclude 3 models: the 87-nt 3_6 SimRNA model because of wrong motif and a large Hamming distance of 14; the 87-nt 3_3 SimRNA model because of a large Hamming distance of 14; and the 144-nt 3_3 RNAComposer model because of incorrect motif and a large Hamming distance of 16. Thus, 23 viable models remain, and are subjected to microsecond MD simulations and further validations as described below.

2.2 MD trajectory convergence

To examine the convergence of the 23 MD trajectories, we first check the system density, which remains at steady levels for all (Supplementary Fig. 2). Second, we check if the FSE RMSD has reached a steady state or plateau, and those with significant fluctuations in the latter half simulations were extended (Supplementary Fig. 3). Six simulations were extended and reached stable states subsequently: 77-nt 3_6 Vfold3D for 0.5 μs; 144-nt 3_6 Farfar2 for 0.25 μs; 77-nt 3_3 RNAComposer, Vfold3D, and SimRNA for 0.25 μs; and 77-nt 3_5 iFoldRNA for 0.25 μs.

Third, since RMSD is a coarse evaluation of structural variability, and low RMSD does not necessarily indicate stable base interactions, we also calculate eRMSD using Barnaba[9] to measure the distance between two 3D structures by considering the relative positions and orientations of their nucleobases[10]. The evolution of eRMSDs over the trajectories is shown in Supplementary Fig. 4, and in all cases, eRMSD maintains a steady state over the last 500 ns.

Finally, we check the simulation stability by monitoring the evolution of the radii of gyration and the number of hydrogen bonds. All MD simulations achieve steady plateaus for the radius of gyration (Supplementary Fig. 5). The cumulative number of hydrogen bonds is counted and plotted against the residue number. The number increases in the $5'$ strand of a stem, and decreases in the $3'$ strand. These mountain-like plots show consistent patterns over simulations for all the systems (Supplementary Fig. 6).

2.3 MD trajectory structure validation

As all MD trajectories for the 23 acceptable candidate models are stable and convergent, we perform additional validation tests. We subject the (equilibrated) start, middle, and end MD structures, as well as the cluster center structure (see Section 4 for clustering details) to the following criteria (see Supplementary Table 2):

1. If the model fails to maintain the correct motif (3_6, 3_3, or 3_5) during the simulation, it is rejected (red).
2. If the Hamming distance between the SHAPE and the model's 2D structure is > 10, the model receives a warning (orange), which makes it less likely to be chosen as the representative structure.
3. For the MD end and cluster center structures, we perform all-atom contact analysis using MolProbity[11], which checks steric clashes, RNA sugar puckers, and RNA backbone conformations. If the structure has a clashscore > 5 (number of steric clashes that overlap $\geq$0.4 Å per thousand atoms), it receives a warning (orange).

After these validations, we exclude 4 models because of wrong motif (see Supplementary Table 2): the 87-nt 3_6 Vfold3D, the 77 and 87-nt 3_3 RNAComposer, and the 87-nt 3_3 Vfold3D. Hence, 19 models remain.

Supplementary Table 1: Wildtype FSE initial 3D model validation. Each model is checked for motif and 2D structure consistency with SHAPE-directed 2D input structure. Models with wrong motif or Hamming distance >10 are rejected and highlighted in red; otherwise, accepted and highlighted in green.

Conformer	Program	2D structure	Motif	Hamming distance
77-nt 3_6	SHAPE	......(((((((((...[[[[[[)))))))))(((((((((.........))).))))))..]].]]]]...		
	RNAComp	.......((((((((...[[[[[[.)))))))).((((((((.........))).))))))...].]]]]...	Yes	4
	iFoldRNA	......(((((((((...[[[[[[.))))))))).((.((((.........))).)).))....].]]]]...	Yes	6
	SimRNA	......(((((((((...[[[[[[)))))))))((((((((((....)).))).))))))..]].]]]].]..	Yes	6
	Vfold3D	......((((((((....[[[[[[..))))))))((((((((.........))).))))))...].]]]]...	Yes	4
87-nt 3_6	SHAPE	(((....)))........((((((((.(([[[[[[)))))))))).((((((((.........))).))))))....]]]]...		
	RNAComp	(((....)))........((((((((.(([[[[[[)))))))))).(((((((.........))).)))))......]]]]...	Yes	2
	iFoldRNA	.((....)).........((((((((..(.[[[[).)))))))).((((((((.........))).))))))....]]]]....	Yes	6
	SimRNA	((((..))))..((..(((((((((.(([[[[[[)))))))))))((((((((((....)).))).))))))..)).]]]].]..	No	14
	Vfold3D	(((....)))........((((((((.(([[[[.)))))))))).((((((((.........))).))))))......]]]]...	Yes	2
144-nt 3_6	SHAPE	..(((.((((....)))).)))....((((....))))......((((((((...[[[[[..))))))))((((((((((.........))).)))))))....]]]]].....((.((((.........)))).)).....		
	RNAComp	..(((.((((....)))).)))....(((......)))......((((((((...[[[[[..))))))))(.((((((((.........))).))))).)....]]]]].....((.((((.........)))).)).....	Yes	4
	iFoldRNA	..(((.((((....)))).)))....((((....))))......((((((((...[[[[[..))))))))(.((((((((..(....).))).))))).)....]]]]]....(((..(.(.........).)..)).)...	Yes	10
	Farfar2	..(((.(((((..))))).)))...(((((....)))))......((((.((((...[[[[[..)))).))))(((((((((.........))).)))))))....]]]]]...(.((.((((..(....).)))).)).)...	Yes	10

**77-nt 3_3**	**SHAPE**	[[[[...((((((((.((.]]]]))))))))))).((((((((.........))).))))))............		
	**RNAComp**	[[[....((((((((.((..]]]))))))))))).(((.(((((.........))).)).)))............	Yes	4
	**iFoldRNA**	[[[[...((((((((.....]]]]...)))))))((((.(((((.........))).)).))))...........	Yes	10
	**SimRNA**	[[[[...((((((((.((.]]]]))))))))))((((((((((((....)).))).)))))))...........	Yes	6
	**Vfold3D**	[[[.....((((((((.((..]]]))))))))))..((((((((.........))).))))))............	Yes	4
**87-nt 3_3**	**SHAPE**	(((((...[[[[[....((((((((.....]]]]]))))))))))((((((((((.........))).)))))))...))))))....		
	**RNAComp**	((.(......[[[....((((((((.....]]]..))))))))((((((((((.........))).)))))))....).))....	Yes	8
	**iFoldRNA**	(((.(...[[[[[.....(((((((......]]]]].))))))).((((((((((.........))).)))))))...).)))....	Yes	6
	**SimRNA**	(((((..([[[[[[)..(((((((......]]]]]]..)))))))(.((((((((((....)).))).))))).)...)))))....	Yes	14
	**Vfold3D**	(.(((...[[.......(((((.(((........]]))).))))((((((((((.........))).)))))))...))).)....	Yes	10
**144-nt 3_3**	**SHAPE**	..(((.((((....)))).)))(((..(((((...[[[[[....((((((((.....]]]]]))))))))).((((((((.........<br>))).)))))))....)))))..))).((.((((.........)))).))....		
	**RNAComp**	..(((.((((....)))).)))(((..(..((............((((((((..........)))))))).(((.(((((.........)))<br>.)).)))....))..)..))).((.((((.........)))).))....	No	16
	**iFoldRNA**	..(((.((((....)))).)))((...(((((...[[[......((((((((........]]].)))))))((((((((((.........)<br>)).)))))))...)))))...)).((.((((.........)))).))....	Yes	10
	**Farfar2**	..(((.((((....)))).)))(((..(((((...[[[[[[...(((((((((....]]]]]]))))))))).((((((((..((..)<br>).))).))))))....)))))..))).((.((((.........)))).))....	Yes	6
**77-nt 3_5**	**SHAPE**	(((((.((((((((((.((.....)))))))))))((((((((.........))).))))))......)))))..		
	**RNAComp**	(((((.((((((((((.((.....)))))))))))((((((((.........))).))))))......)))))..	Yes	0
	**iFoldRNA**	.((((..((((((.((.((.....))))).))))))..((.(((((.........))).)).)).......)))...	Yes	10
	**SimRNA**	(((((((((((((((.((.....)))))))))))).((((((((((....)).))).))))))...)).)))))..	Yes	8
	**Vfold3D**	(((((.((((((((((..(.....).))))))))))((((((((.........))).))))))......)))))..	Yes	2

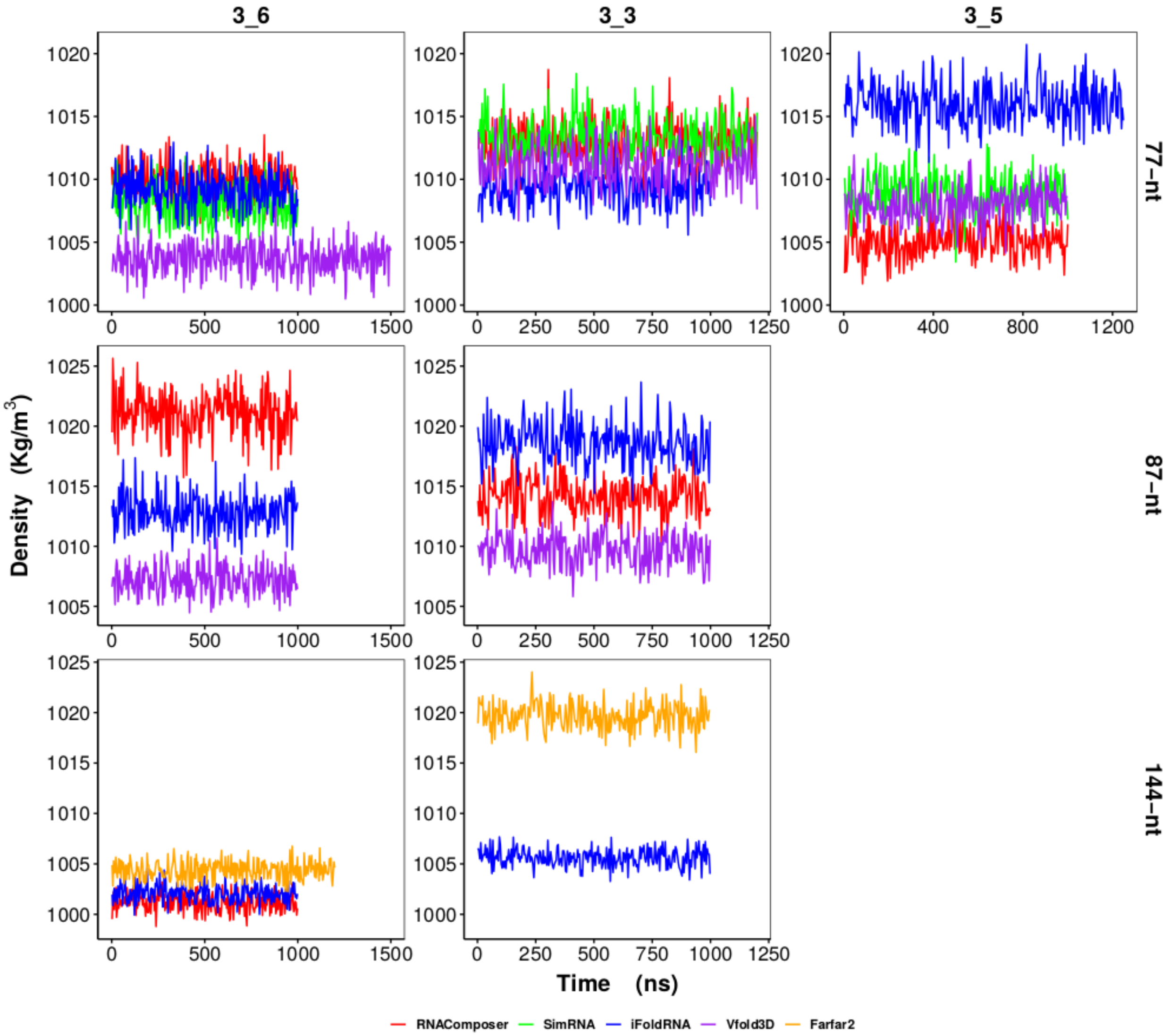

Supplementary Figure 2: Time evolution of system density in the 23 wildtype FSE MD simulations.

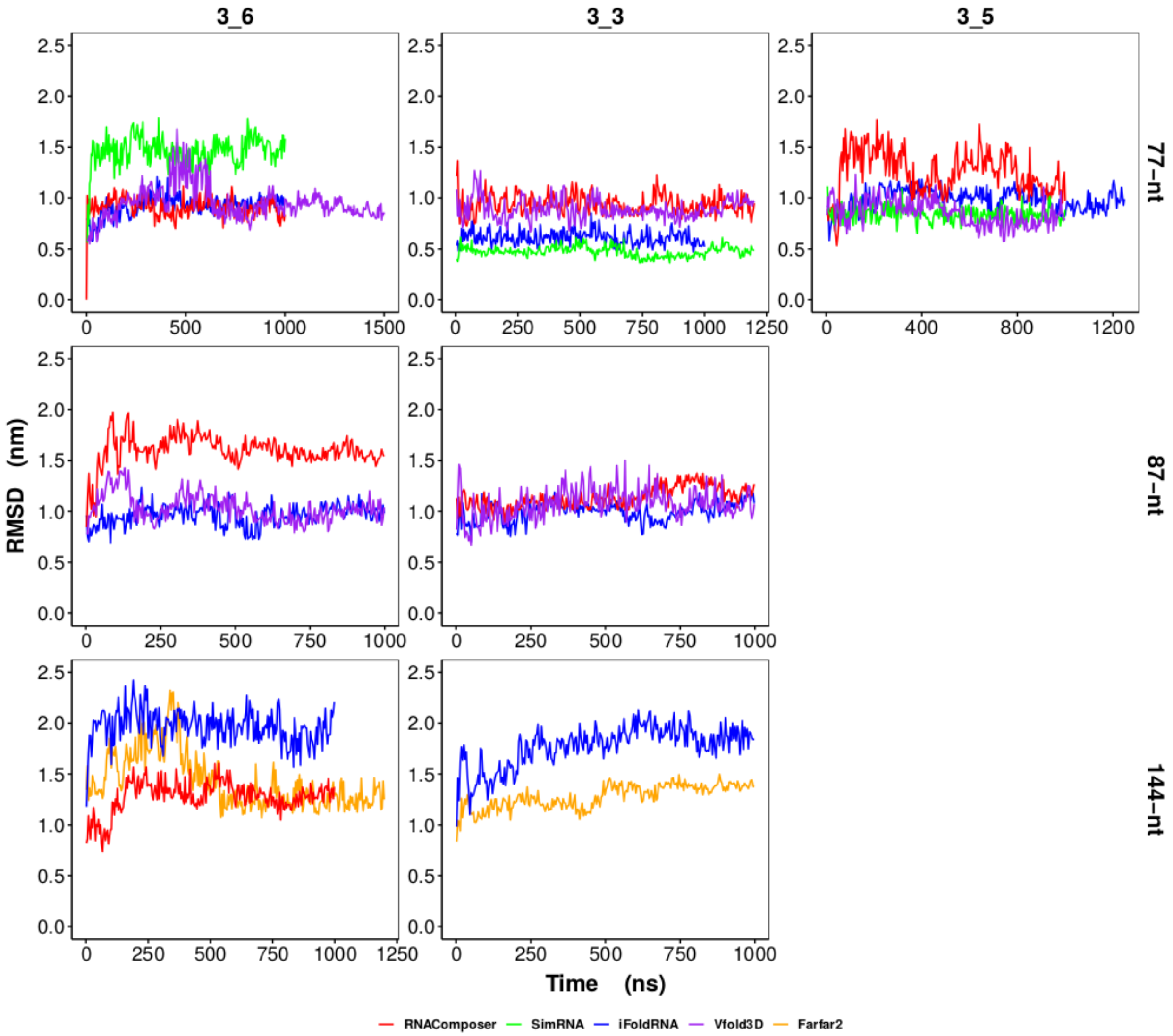

Supplementary Figure 3: Time evolution of RMSD in the 23 wildtype FSE MD simulations.

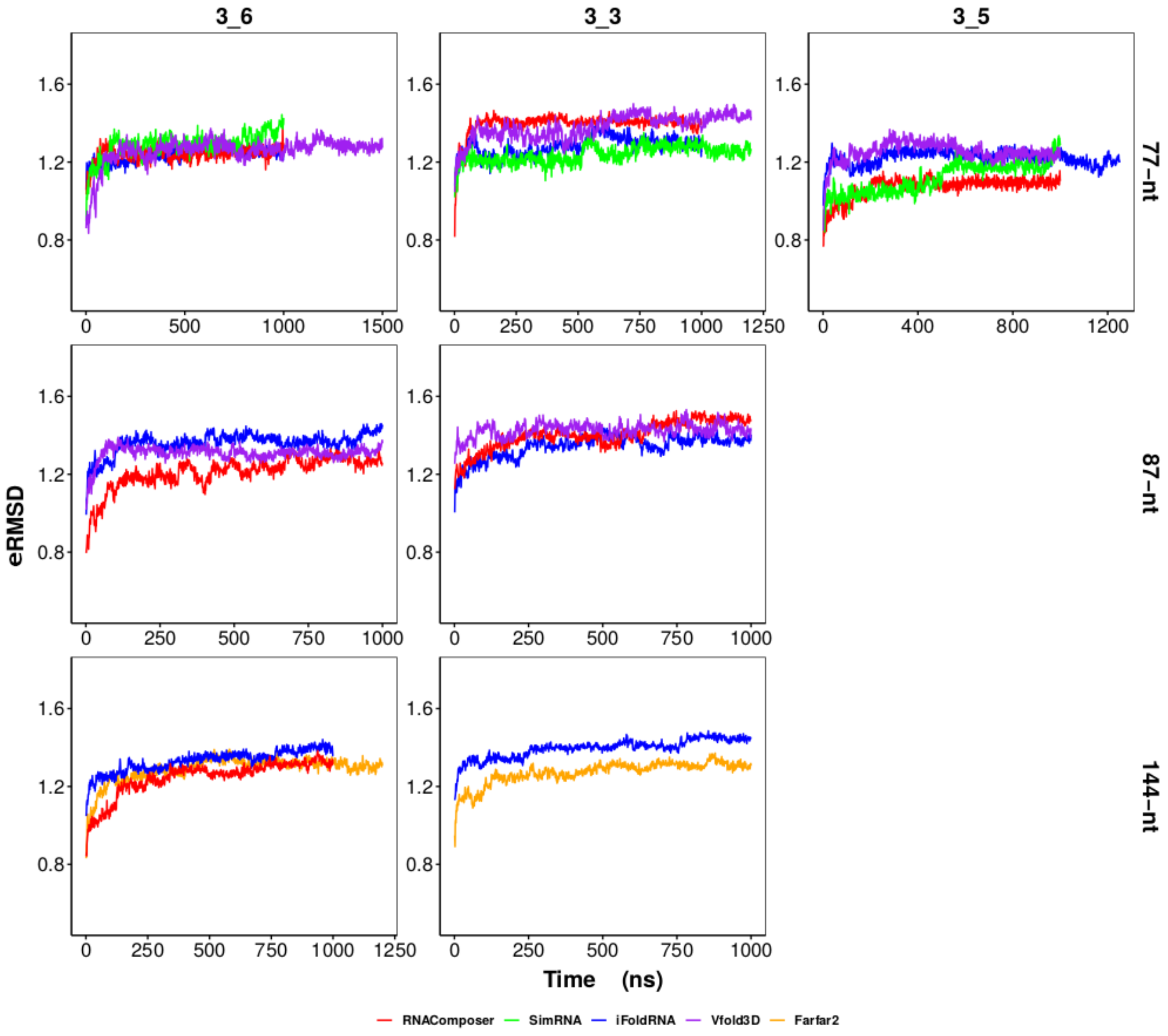

Supplementary Figure 4: Time evolution of eRMSD in the 23 wildtype FSE MD simulations.

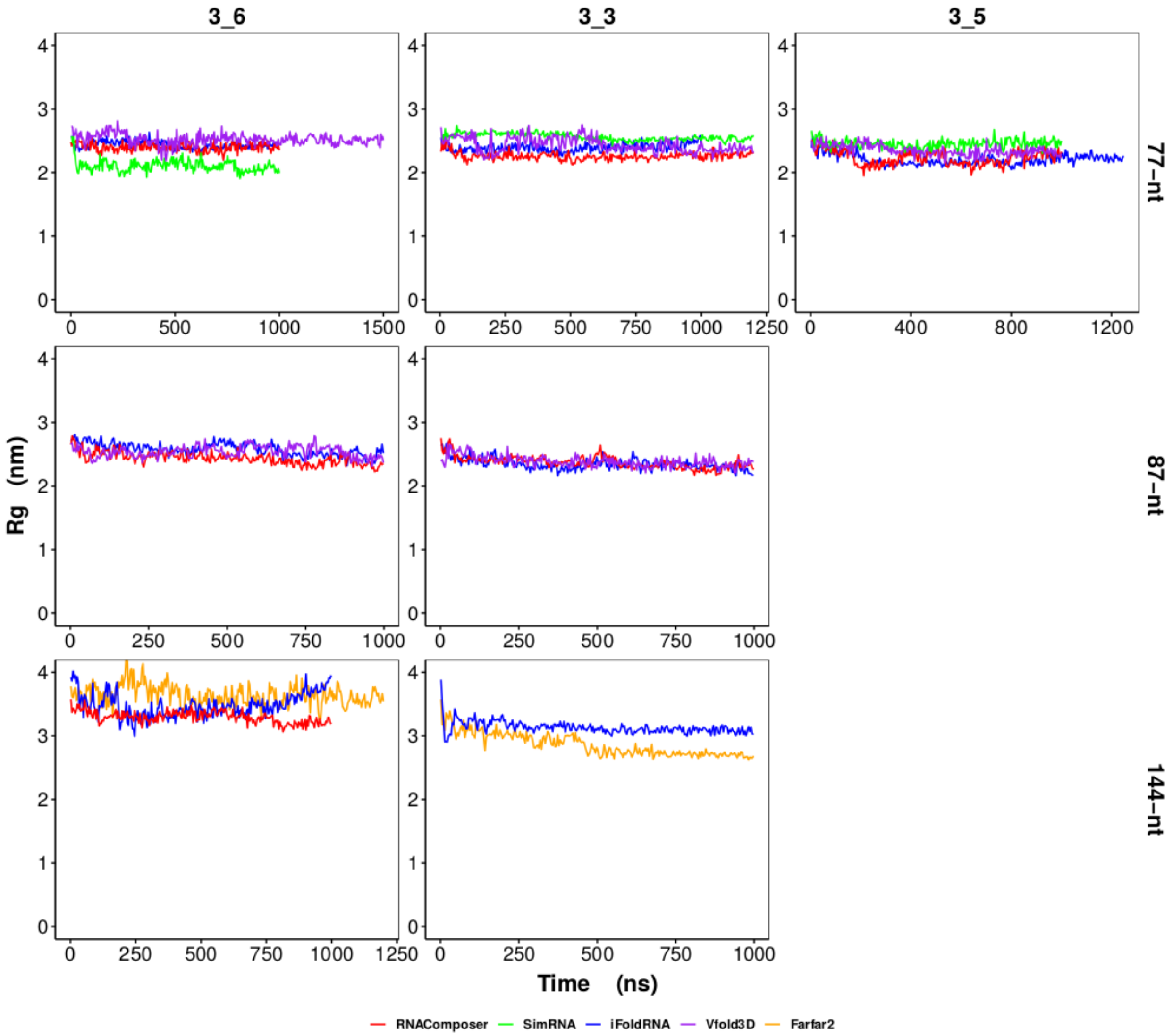

Supplementary Figure 5: Time evolution of the radius of gyration in the 23 wildtype FSE MD simulations.

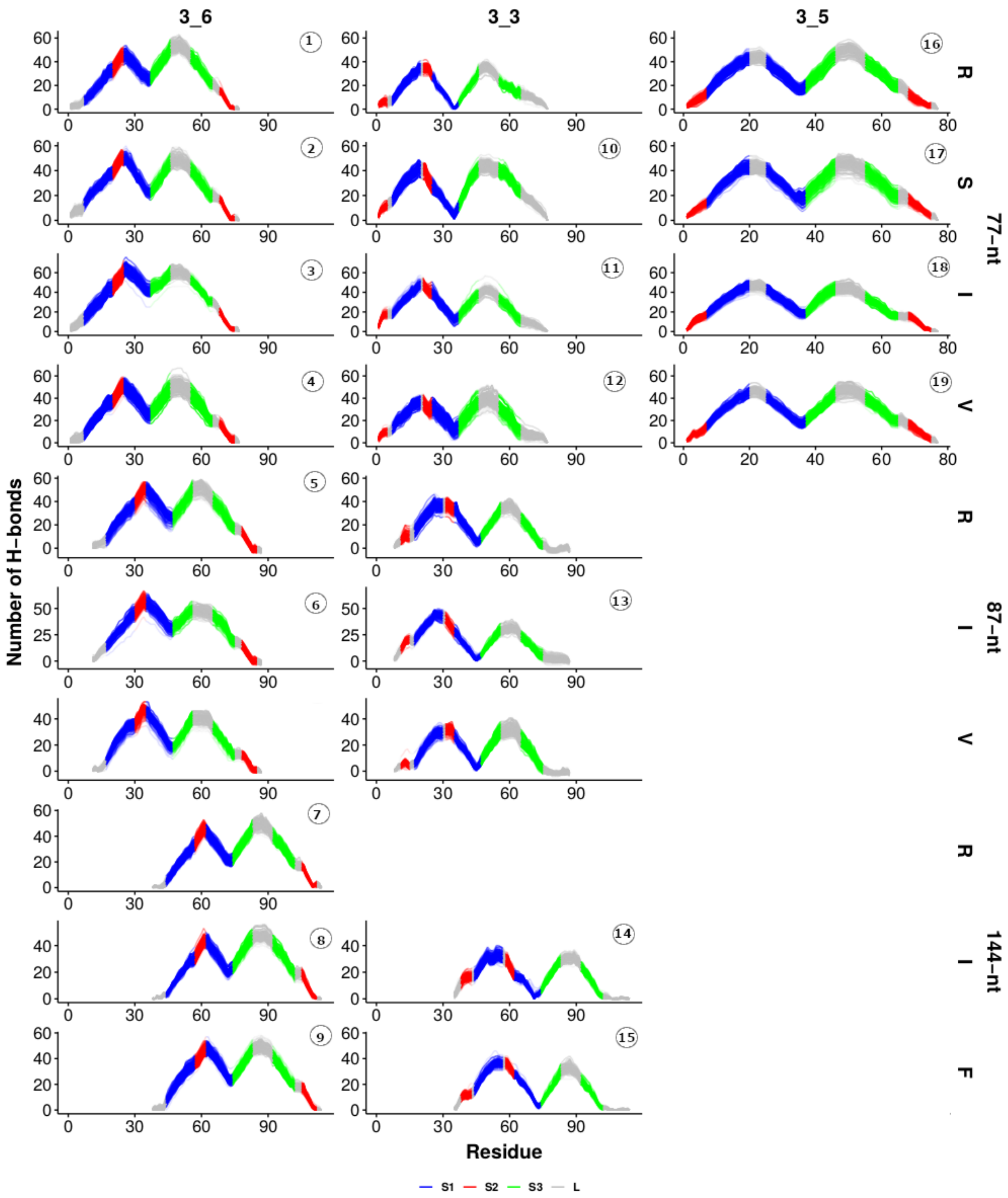

Supplementary Figure 6: Hydrogen bonds in the 23 wildtype FSE MD simulations. The cumulative number of hydrogen bonds by residue is calculated, with S1 residues in blue, S2 in red and S3 in green. Data for analysis are extracted from MD structures every 100 ns. Trajectory numbers from Table 1 in the paper are labeled for reference. Those without numbers were rejected.

Supplementary Table 2: Wildtype FSE MD model validation. For each MD trajectory, the start, middle, and end frames, as well as the cluster center structure are checked for motif and 2D structure consistency with SHAPE-directed 2D input structure. Clashscores are calculated using MolProbity [11], and 3_6 models are aligned with the four experimental structures (Jones et al., PDB: 7LYJ [12]; Roman et al., PDB: 7MLX [13]; Bhatt et al., PDB: 7O7Z [14]; Zhang et al., PDB: 6XRZ [15]) using PyMol *align* [16]. Trajectories that fail to maintain the correct motifs are rejected (red), and those with Hamming distances >10 or clashscores >5 are noted but still retained (orange).

Conformer	Program	Timestep	2D structure	Motif	Hamming distance	RMSD from reference (Å)	Clash score
77-nt 3_6	SHAPE		......(((((((((...[[[[[[[)))))))))((((((((.........))).))))))..]].]]]]]...				
	RNAComp	MD start	........((((((((...[[[[[[.))))))))..((((((((.........))).))))))...].]]]]]...	Yes	6		
		MD mid	........(.((((((...[[[[[..)))))))...((((((((.........))).))))))......]]]].]..	Yes	12		
		MD end	........(.((((((....[[[[.[)))))))...((((((((.........))).)))))).]..]]]]....	Yes	10	8.46 (Jones)<br>6.55 (Roman)<br>10.86 (Bhatt)<br>10.60 (Zhang)	2.84
		Cluster	........(..(((((....[[[[..))))).)...((((((((.........))).))))))......]]]]....	Yes	14	7.05<br>5.50<br>11.27<br>10.93	1.62
	iFoldRNA	MD start	.......(((((((((...[[[[[[.)))))))))..((((((((.........))).)))))....].]]]]]...	Yes	6		
		MD mid	.....(((((((((((...[[[[[[[)))))))))))((((((((.........))).)))))...]].]]]]]...	Yes	3		
		MD end	.......((.((((((...[[[[[[[))))))).))..((((((((.........))).)))))...]].]]]]]...	Yes	6	4.00<br>4.77<br>12.51<br>13.31	3.65
		Cluster	.....(((((((((((...[[[[[[[)))))))))))((((((((.........))).)))))...]].]]]]]...	Yes	3	3.67<br>4.81<br>13.00<br>13.17	2.03
	SimRNA	MD start	......(((((((((...[[[[[[[)))))))))((((((((((....)).))).))))))..]].]]]].]..	Yes	6		
		MD mid	......((((.(((.(....[[[[.[).))).)))).((((.(((.........)))..))))...]..]]]]....	Yes	12		
		MD end	.......((((((((.(..[[[[.)))))))))..((((((((.........))).)))))......]]]]....	Yes	11	13.33<br>13.30<br>11.52<br>11.69	3.25
		Cluster	......((((((((((.(..[[[[.))))))))))).((((.(((.........)))..))))......]]]]....	Yes	11	13.78<br>13.47<br>11.48<br>12.62	2.43

	**Vfold3D**	MD start	......((((((((....[[[[[..))))))))((((((((.........))).))))))...].]]]]...	Yes	4		
		MD mid	......(((((((((...[[[[[..)))))))))).(((((((.(....)..))).)))))......]]]]...	Yes	8		
		MD end	......(((((((((....[[[[..))))))))))((((((((.(....)..))).))))))).....]]]]....	Yes	8	4.48<br>4.73<br>12.12<br>12.59	4.06
		Cluster	......(((((((((....[[[[..))))))))))((((((((.(....)..))).))))))).....]]]]....	Yes	8	3.65<br>4.93<br>12.68<br>12.86	1.62
**87-nt 3_6**	**SHAPE**		(((....)))........((((((((.(([[[[[))))))))))).((((((((.........))).))))))......]]]]]...				
	**RNAComp**	MD start	(((....)))......(((((((((.(([[[[[)))))))))))((((((((.........))).)))))).....]]]]]...	Yes	2		
		MD mid	((......))......(..((((((((.((.[[[[)))))))))))..((((((((.........))).)))))......]]]]....	Yes	8		
		MD end	(((....)))........((((((((.((.[[[[)))))))))))...((((((((.........))).)))))......]]]]....	Yes	6	7.48<br>8.46<br>11.00<br>10.14	3.24
		Cluster	(((....)))......(..((((((((.((.[[[[)))))))))))..((((((((.........))).)))))......]]]]....	Yes	6	7.79<br>8.16<br>9.88<br>10.15	5.04
	**iFoldRNA**	MD start	.((....))..........(((((.(.((.[[[[))).)))))....((((((((.........))).)))))......]]]]....	Yes	12		
		MD mid	...(....)........((((((((..(.[[[[).)))))))).((((((((.........))).))))))......]]]]....	Yes	10		
		MD end	..................((((((((...(.[[[[)..))))))..((((((((.............).)))))).....]]]]....	Yes	18	4.30<br>6.85<br>15.60<br>13.97	2.16
		Cluster	................((((((((..(.[[[[).)))))))).(((((.(((.........)))..))))).....]]]]....	Yes	12	3.99<br>7.07<br>15.80<br>14.25	2.16
	**Vfold3D**	MD start	(((....)))......(((((((((.(([[[[.)))))))))))((((((((.........))).)))))).....].]]]...	Yes	4		
		MD mid	((......))......((((((((((....[[[...))))))))))..(((((((.........))).))))........]]]....	Yes	16		
		MD end	((......))......((((((((((....[[[...))))))))))..(((((((.........))).))))........]]]....	Yes	16	11.20<br>8.10<br>14.64<br>11.70	5.04

		Cluster	................((((((((..(.[[[[).)))))))).((((.(((.........)))..)))))......]]]]....	No	12	11.25<br>5.52<br>14.34<br>8.81	3.6
144-nt 3_6	**SHAPE**		..(((.((((....)))).)))....((((....))))......((((((((...[[[[[..))))))))(((((((((......<br>...))).)))))))....]]]]].....((.((((.........)))).))....				
	**RNAComp**	MD start	..(((.((((....)))).)))....(((......)))......((((((((...[[[[[..))))))))(((((((((.......<br>..))).)))))))....]]]]].....((.((((.........)))).)).....	Yes	2		
		MD mid	...((.((((....)))).)).....(((......))).......((((((((..(.[[[[).))))))))...((((((((........<br>))).)))))......]]]]......((.((((.........)))).)).....	Yes	14		
		MD end	...((.(((......))).)).....(((......))).......((((((((..(.[[[[).))))))))...((((((((........<br>))).)))))......]]]]......((.((((.........)))).)).....	Yes	16	5.55<br>7.45<br>10.88<br>11.60	3.26
		Cluster	..(((.((((....)))).)))....(((......))).......((((((((..(.[[[[).))))))))...((((((((........<br>.))).)))))......]]]]......((.((((.........)))).)).....	Yes	12	4.17<br>6.63<br>12.14<br>11.35	1.74
	**iFoldRNA**	MD start	..(((.((((....)))).)))...(((((....)))))......((((((((...[[[[[..))))))))(((((((((..(...<br>.).)))).)))))))....]]]]]....(((..(((.........)))..)).)...	Yes	8		
		MD mid	..(((.((((....)))).)))...((((......))))......((((((((.(.[[[[[.))))))))).((((((((((.......<br>..))).)))))))....]]]].]....((.((((.........)))).)).....	Yes	10		
		MD end	..(((.(((......))).)))...(((((....)))))......((((((((...[[[[[..))))))).((((((((((.......<br>..))).)))))))....]]]].]...(((.((((.........)))).)).)...	Yes	10	2.82<br>5.02<br>16.14<br>13.25	4.35
		Cluster	..(((.(((......))).)))...(((((....)))))......((((((((.(.[[[[[.))))))))).((((((((........<br>...)).)))))))....]]]].]..((((.((((.........)))).)).))..	Yes	16	3.55<br>7.42<br>12.45<br>12.31	2.18
	**Farfar2**	MD start	..(((.(((((..))))).)))...(((((....)))))......((((((((...[[[[[..))))))))(((((((((......<br>....))).)))))))....]]]]]...(.((.((((..(....).)))).)).)...	Yes	8		
		MD mid	..(((.(((......))).)))......(......).......((((((((((...[[[[[..))))))))).((((((((.........<br>))).)))))......]]]]].....(..((((.........))))..).....	Yes	15		
		MD end	..(((.((((....)))).)))...(..(......)..)....((((((((....[[[[[...))))))))).((((((((.........<br>))).)))))......]]]]].....((.((((.........)))).)).....	Yes	15	7.05<br>3.50<br>11.82<br>11.72	3.05
		Cluster	..(((.(((......))).)))...(..(......)..)....((((((((....[[[[[...))))))))).((((((((.........<br>))).)))))......]]]]]...((((.((((.........)))).))..).)	Yes	21	8.37<br>4.10<br>12.34<br>10.57	1.52
77-nt 3_3	**SHAPE**		[[[[...((((((((((.((.]]]]))))))))))).(((((((((.........))).)))))).............				

	**RNAComp**	MD start	[[[....((((((((.((..]]])))))))))).((((((((.........))).))))).............	Yes	4		
		MD mid	.......((((((((.((.....))))))))))((((((((((.........)))[])))))).........]	No	12		
		MD end	.......((((((((.((.....))))))))))((((((((((.........)))[])))))).........]	No	12		1.12
		Cluster	.......((((((((.((.....))))))))))((((((((((.........)))[])))))).........]	No	12		2.43
	**iFoldRNA**	MD start	[[[[...(((((((((....]]]]..)))))))))(((.(((((.........))).)).))))..........	Yes	10		
		MD mid	[[[[...((((((((.((.]]]])))))))))))((((((((((.........))).)))))))..........	Yes	2		
		MD end	[[[[...((((((((.((.]]]])))))))))))).((((((((.........))).))))))..........	Yes	0		2.43
		Cluster	[[[....((((((((.((.{]]])))))))))))((((((.(((.........)))..)))))).........}.	Yes	7		2.43
	**SimRNA**	MD start	[[[[...((((((((.((.]]]])))))))))))((((((((((((....)).))).)))))))..........	Yes	6		
		MD mid	[[.[...((((((((.(([..]])))))))))))(((((((.(..........)..)))))))..........	Yes	10		
		MD end	[[.[...(((((((..(([..]])).))))))))((((.((.(..........)..)).))))..........	Yes	14		5.27
		Cluster	[[.[...((((((((.(([..]]))))))))))))((((.((.(..........).[)).)))).........].	Yes	14		0.81
	**Vfold3D**	MD start	[[[.....((((((((.((..]]])))))))))))..((((((((((.........))).))))))..........	Yes	4		
		MD mid	[[.....(((.(((((.....]]...))))).)))...((((((((.........))).))))..............	Yes	14		
		MD end	[[.....(((.(((((.....]]...))))).)))(((((((((((.........))).)))))))..........	Yes	12		2.03
		Cluster	[[.....(((.(((((.....]]...))))).))).((((((((((.........))).))))))..........	Yes	10		2.84
**87-nt 3_3**	**SHAPE**		(((((...[[[[[....(((((((((.....]]]]])))))))))((((((((((.........))).)))))))...)))))....				
	**RNAComp**	MD start	((((........[....(((((((((.....]....))))))))((((((((((.........))).)))))))....))))....	No	10		
		MD mid	..((............((((((((((..........))))))))..(((((((((.........))).)))))......))......	No	20		
		MD end	...((..........((((((((((..........))))))))))((((((((((.........))).))))))....)).......	No	19		3.24

		Cluster	..((...........(((((((((..........)))))))))((((((((.........))).)))))).....))......	No	19		1.44
	iFoldRNA	MD start	(((((...[[[[[....(((((((((.....]]]]])))))))))((((((((((.........))).)))))))...))))).....	Yes	0		
		MD mid	...(....[[[[[....((((((((......]]]]].)))))))).(((.(((((.........))).)).)))......).......	Yes	14		
		MD end	....(...[[[[[.....((((((((.....]]]]])))))))).(((((((((.........))).)))))))....).......	Yes	10		0.72
		Cluster	...(....[[[[[....(((((((((.....]]]]]))))))))).((((((((.........))).))))))......).......	Yes	10		1.8
	Vfold3D	MD start	(((((...[.[[.[....((((((((....].]].]))))))))).(((((((((.........))).)))))))...)))))....	Yes	8		
		MD mid	((((..............((((((((..........)))))))).(((((((((.........))).)))))))....)))).....	No	14		
		MD end	(..(..............((((((((..........)))))))).(((((.((((.........))).).)))))....)..).....	No	20		2.16
		Cluster	...(..............((((((((..........)))))))).(((((((((.........))).)))))))....).......	No	20		1.8
144-nt 3_3	SHAPE		..(((.((((....)))).)))(((..(((((...[[[[[....(((((((((.....]]]]])))))))))).(((((((((..........))).))))))....)))))..))).((.((((.........)))).))....				
	iFoldRNA	MD start	..(((.((((....)))).)))(((..(((((...[[[[......((((..(......]]]])..))))).(((((((((........))).)))))))...)))))..))).((.((((.........)))).)).....	Yes	10		
		MD mid	..(((.((((....)))).)))(((..........[[[[......(((((.........]]]]...)))))..((((((((.........))).))))))...........))).((.((((.........)))).)).....	Yes	20		
		MD end	...((.((((....)))).)).(((...........[[[......(((((.........]]]....)))))..(((((((((.........))).))))))...........))).((.((((.........)))).)).....	Yes	24		2.61
		Cluster	...(..((((....))))..).(((..........[[[[......(((((.........]]]]...)))))..(((((((((.........))).))))))...........))).((.((((.........)))).)).....	Yes	24		2.18
	Farfar2	MD start	..(((.((((....)))).)))(((..(((((...[[[[[[..((((((((.]..]]]]]])))))))))).(((((((((.........))).))))))....)))))..))).((.((((.........)))).)).....	Yes	4		
		MD mid	..(((.((((....)))).)))(((...((......[[[....(((((((((......]]].)))))))))((((((((.........))).))))))......))...))).((.((((.........)))).)).....	Yes	12		
		MD end	..(((.((((....)))).)))(((.....((.....[[[....(((((((((......]]].)))))))))((((((((.........))).))))))......))....))).((.((((.........)))).)).....	Yes	12		3.7
		Cluster	...((.((((....)))).)).(((....(......[[[....((((((.(((......]]].))).))))))(((((((((.........))).))))))......)....))).((.((((.........)))).)).....	Yes	18		2.39
77-nt 3_5	SHAPE		(((((.((((((((((.((.....)))))))))))((((((((.........))).))))))......)))))..				

	**RNAComp**	MD start	((.(((((((((((.((.....))))))))))(((((((((.........))).))))))..)))).))...	Yes	9		
		MD mid	....((.(((((((((.((.....)))))))))).((((((((.........))).))))))....))......	Yes	14		
		MD end	....((((((((((((.((.....)))))))))).((((((((.........))).))))))...)))......	Yes	14		1.62
		Cluster	....((((((((((((.((.....)))))))))).((((((((.........))).))))))...)))......	Yes	14		1.62
	**iFoldRNA**	MD start	(((((.((((((((((.((.....))))))))))).((((((((.........))).))))).......)))))..	Yes	2		
		MD mid	(.(((..(((((((((..(.....).))))))))).((((((((...........)).))))))......))).)..	Yes	8		
		MD end	((((..(((((((((..(.....).)))))))))..(((((((...........)).))))).......)))))..	Yes	8		3.65
		Cluster	(.((.(((((((((((..(.....).))))))))))(((((((...........)).))))))......))).)..	Yes	8		0.41
	**SimRNA**	MD start	((((((.(((((((((.((.....)))))))))).((((((((.((....))..)).))))))......))))))..	Yes	10		
		MD mid	((((((..((((((((..........))))))))..((((((((.((....))..)).)))))).....))))))..	Yes	16		
		MD end	(((...(.((((((((..........)))))))).)(((((.((...........))..)))))........)))..	Yes	16		3.65
		Cluster	(((.....((((((((..........))))))))..((((((((.(......)..)).))))))........)))..	Yes	16		3.65
	**Vfold3D**	MD start	(((((.((((((((((..(.....).))))))))))(((((((((.........))).))))))......)))))..	Yes	2		
		MD mid	(((..(((((((((((..(.....).)))))))))).((((((((.........))).)))))........).)))..	Yes	8		
		MD end	(((..(((((((((((..(.....).)))))))))).((((((((.........))).)))))........).)))..	Yes	8		4.06
		Cluster	(((..(((((((((((..(.....).))))))))))((((((((((((....)).))).))))))......).)))..	Yes	10		2.03

3 MD/Experiment comparison and representative model selection

We align all the validated 3_6 MD end and cluster center structures with the four available experimental structures using common FSE regions (66-nt Jones et al. crystallography[12], 65-nt Roman et al. crystallography[13], 77-nt FSE segment from the Bhatt et al. mRNA-ribosome Cryo-EM complex[14], 88-nt Zhang et al. Cryo-EM[15]), with RMSD calculated (Supplementary Table 2).

We summarize all the validation and alignment results in Supplementary Table 3. For 77-nt 3_6, Vfold3D receives no warning and has the lowest crystal structure alignment RMSD (3.65 Å) with the Jones et al. model, followed by iFoldRNA (3.67 Å). However, Vfold3D fails to maintain the 3_6 motif at 87-nt, and cannot predict the 144-nt 3_6 due to sequence length limitation. Because the 87 and 144-nt iFoldRNA systems have the lowest RMSDs when aligned to the Jones et al. crystal structure, we choose them as representatives for 3_6. Regarding the alignment with Cryo-EM structures, RNAComposer systems achieve the lowest RMSDs at 77 and 87-nt, and the second lowest at 144-nt, so they are chosen as 3_6 representatives as well.

For 3_3 systems, only iFoldRNA systems maintain the correct motif at all lengths. Moreover, they always receive the least number of warnings. Hence, we choose iFoldRNA systems as the representative structures. For 3_5 systems, both iFoldRNA and Vfold3D receive no warning. From multi-trajectory cluster analysis shown in Fig. 2 of the main manuscript, we find they form a compact and an elongated 3_5 junction. Hence, we choose both as representative cases.

Supplementary Table 3: Summary table for the wildtype model validations. For rejected models, we specify step number (initial or MD validation) and reason for rejection. For accepted models, we specify how many warnings they receive due to large Hamming distances and high clashscores. In addition, we list the best alignment RMSDs between our 3_6 cluster centers and the two crystal structures by Jones et al. (PDB ID: 7LYJ) and Roman et al. (PDB ID: 7MLX), as well as the best alignment with the Cryo-EM structure by Zhang et al. (PDB ID: 6XRZ) and the FSE segment extracted from the Bhatt et al. mRNA-ribosome Cryo-EM complex (PDB ID: 7O7Z). The lowest RMSDs are labeled with asterisk for each length. Trajectory numbers 1-19 refer to labels used in Table 1 with representatives highlighted in yellow.

**Rejected Models**			
**Conformer**	**Program**	**Step**	**Rejection reason**
87-nt 3_6	SimRNA	Initial	Wrong motif, Hamming 14
87-nt 3_3	SimRNA	Initial	Hamming 14
144-nt 3_3	RNAComp	Initial	Wrong motif, Hamming 16
87-nt 3_6	Vfold3D	MD	Wrong motif (cluster)
77-nt 3_3	RNAComp	MD	Wrong motif (MD mid, end, cluster)
87-nt 3_3	RNAComp	MD	Wrong motif (MD start, mid, end, cluster)
87-nt 3_3	Vfold3D	MD	Wrong motif (MD mid, end, cluster)

**Accepted Models**					
**Conformer**	**Program**		**Warnings**	**Crystal RMSD (Å)**	**Cryo-EM RMSD (Å)**
77-nt 3_6	1	RNAComp	1	5.50 (Roman)	10.93* (Zhang)
	2	SimRNA	3	13.47 (Roman)	11.48 (Bhatt)
	3	iFoldRNA	0	3.67 (Jones)	13.00 (Bhatt)
	4	Vfold3D	0	3.65* (Jones)	12.68 (Bhatt)
87-nt 3_6	5	RNAComp	0	7.79 (Jones)	9.88* (Bhatt)
	6	iFoldRNA	3	3.99* (Jones)	14.25 (Zhang)
144-nt 3_6	7	RNAComp	3	4.17 (Jones)	11.35 (Zhang)
	8	iFoldRNA	1	3.55* (Jones)	12.31 (Zhang)
	9	Farfar2	3	4.10 (Roman)	10.57* (Zhang)
77-nt 3_3	10	SimRNA	3		
	11	iFoldRNA	0		
	12	Vfold3D	2		
87-nt 3_3	13	iFoldRNA	1		
144-nt 3_3	14	iFoldRNA	3		
	15	Farfar2	3		
77-nt 3_5	16	RNAComp	3		
	17	SimRNA	3		
	18	iFoldRNA	0		
	19	Vfold3D	0		

4 Wildtype FSE MD clustering analysis

4.1 Conformational sampling heterogeneity

To identify different FSE conformations sampled by the MD simulations, we perform clustering analysis for each of our validated 19 MD trajectories listed in Table 1. Structures from the last 500 ns are extracted every 200 ps, so 2500 structures are used for each trajectory. A cutoff of 2.5 Å is set for the 77-nt and 87-nt systems, and a cutoff of 3.5 Å is set for the 144-nt systems, so that a feasible number of clusters is produced with outlier structures excluded. In Supplementary Fig. 7, we rank the clusters by size, and plot the cumulative fraction of structures contained in the clusters against the number of clusters. We count the number of top clusters that contain 75% of structures in each trajectory (Supplementary Table 4.)

The cluster numbers vary significantly among different trajectories, suggesting that some trajectories sample a wider region than others (Supplementary Fig. 7). Interestingly, the most heterogeneous and homogeneous sampling both occur in the 3_6 iFoldRNA systems, with 85 clusters covering 75% structures for 144-nt trajectory, while only 2 clusters for 77-nt (Supplementary Table 4). Similar observations from 3_3 and 3_5 systems further suggest that the sampling performance is independent of the 3D program and the FSE motif. Moreover, no relation is found between cluster numbers and system lengths. For the 3_6 systems, 144-nt trajectories have more clusters than 77-nt and 87-nt, but for 3_3, 144-nt trajectories have the least numbers of clusters.

4.2 Major conformations sampled

Centers of the top clusters that contain 75% of the structures are superimposed in Supplementary Fig. 8. Overall, the cluster centers of each trajectory align well. The loop regions fluctuate more than the stems.

For the 3_6 pseudoknot, both an L and a linear shape are captured. At 77-nt, trajectories 1 and 2 have the L shape and trajectories 3 and 4 adopt the linear. At longer lengths, all exhibit linear shape except trajectory 5. Compared to the central FSE 3-stem region, the two ends take distinct helical arrangements in different systems, as we can see from trajectories 7-9. Another notable feature of the 3_6 structures is the 5′ threading described in Fig. 4. Here, we find that all L shape 3_6 structures have threading, probably due to a wider ring hole caused by Stem 3 bending (Supplementary Fig. 8). For the linear shape, the non-threaded conformation is preferred.

For the 3_3 pseudoknot, the triplets discussed in Fig. 6 involving all Stem 2 interactions are found in 2 of the 3 trajectories at 77-nt (Supplementary Table 4). The flanking stem SF forms in all systems at 87-nt and 144-nt, which

eliminates alternative Stem 2 interactions and stabilizes the 3_3 pseudoknot.

For the 3_5 junction, two trajectories take an elongated shape, and two others are more compact. The two elongated structures both have Stems 1 and 2 co-axial stacking (Supplementary Table 4).

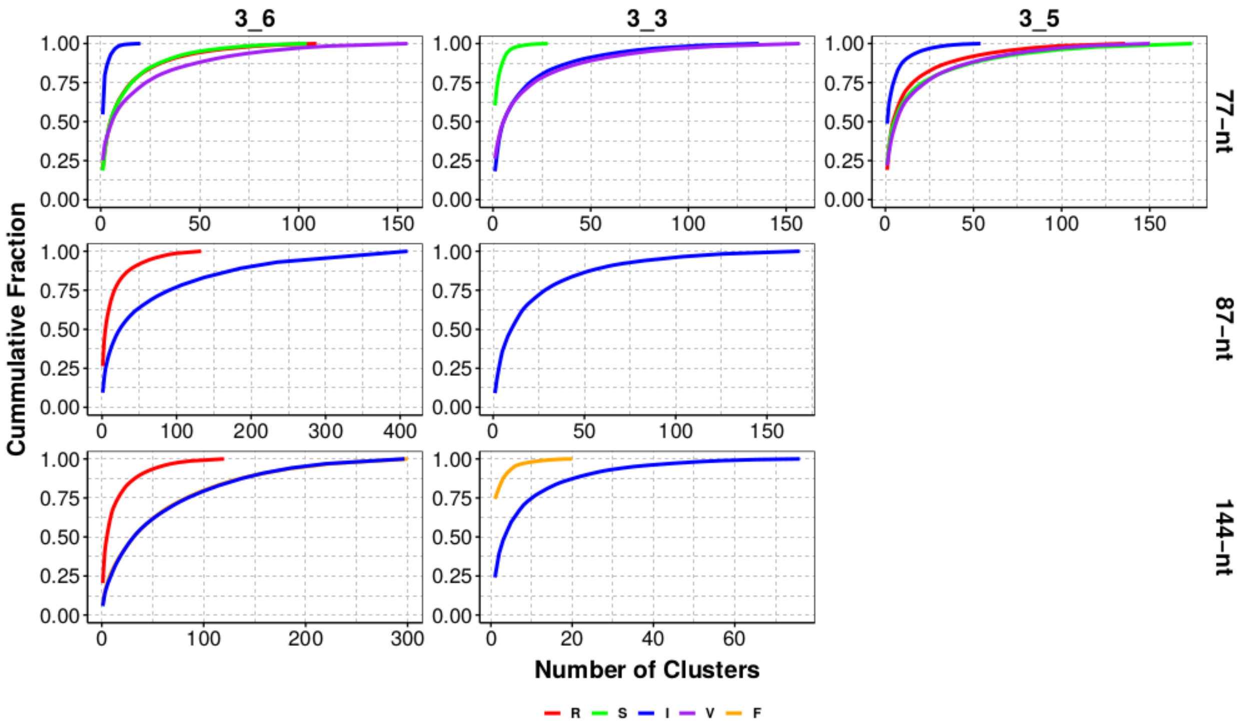

Supplementary Figure 7: Cluster analysis of the FSE MD simulations for the 19 validated structures. Cumulative fraction of the structures is calculated as the number of clusters increases. The clusters are ranked from the largest to the smallest in size. To make sure that a feasible number of clusters exist in all the systems of the same length, a cutoff is defined to be 2.5 Å for 77 and 87-nt, or 3.5 Å for 144-nt.

Supplementary Table 4: Conformational details of the validated wildtype systems. The trajectories are numbered following Table 1. Representative systems are highlighted in yellow. The number of clusters needed to capture 75% of the MD structures is listed. For 3_6, we indicate if the 5′ end threads through the ring (formed by 3′ strand of Stem 1, and the Stem 1/3 and 2/3 junctions, see Fig. 4), and whether the structure holds the L or linear shape. For 3_3, we check if similar triplets seen in Fig. 6 are formed by Stem 2 with the 3′ end, and whether the flanking stem SF forms. For 3_5, we indicate the co-axial stacking and the shape.

**3_6 Systems**			
Trajectory	Clusters	Threaded 5′ end	L or linear shape
1. 77-nt R	16	Yes	L
2. 77-nt S	16	Yes	L
3. 77-nt I	2	No	Linear
4. 77-nt V	23	Yes	Linear
5. 87-nt R	3	Yes	L
6. 87-nt I	38	No	Linear
7. 144-nt R	17	No	Linear
8. 144-nt I	85	No	Linear
9. 144-nt F	83	Yes	Linear
**3_3 Systems**			
Trajectory	Clusters	Stem 2 triplets	Stem SF
10. 77-nt S	3	No	No
11. 77-nt I	19	Yes	No
12. 77-nt V	21	Yes	No
13. 87-nt I	29	No	Yes
14. 144-nt I	11	No	Yes
15. 144-nt F	2	No	Yes
**3_5 Systems**			
Trajectory	Clusters	Co-axial stacking	Elongated or compact
16. 77-nt R	16	S1, S2	Compact
17. 77-nt S	22	S1, S2	Elongated
18. 77-nt I	5	S1, S3	Compact
19. 77-nt V	23	S1, S2	Elongated

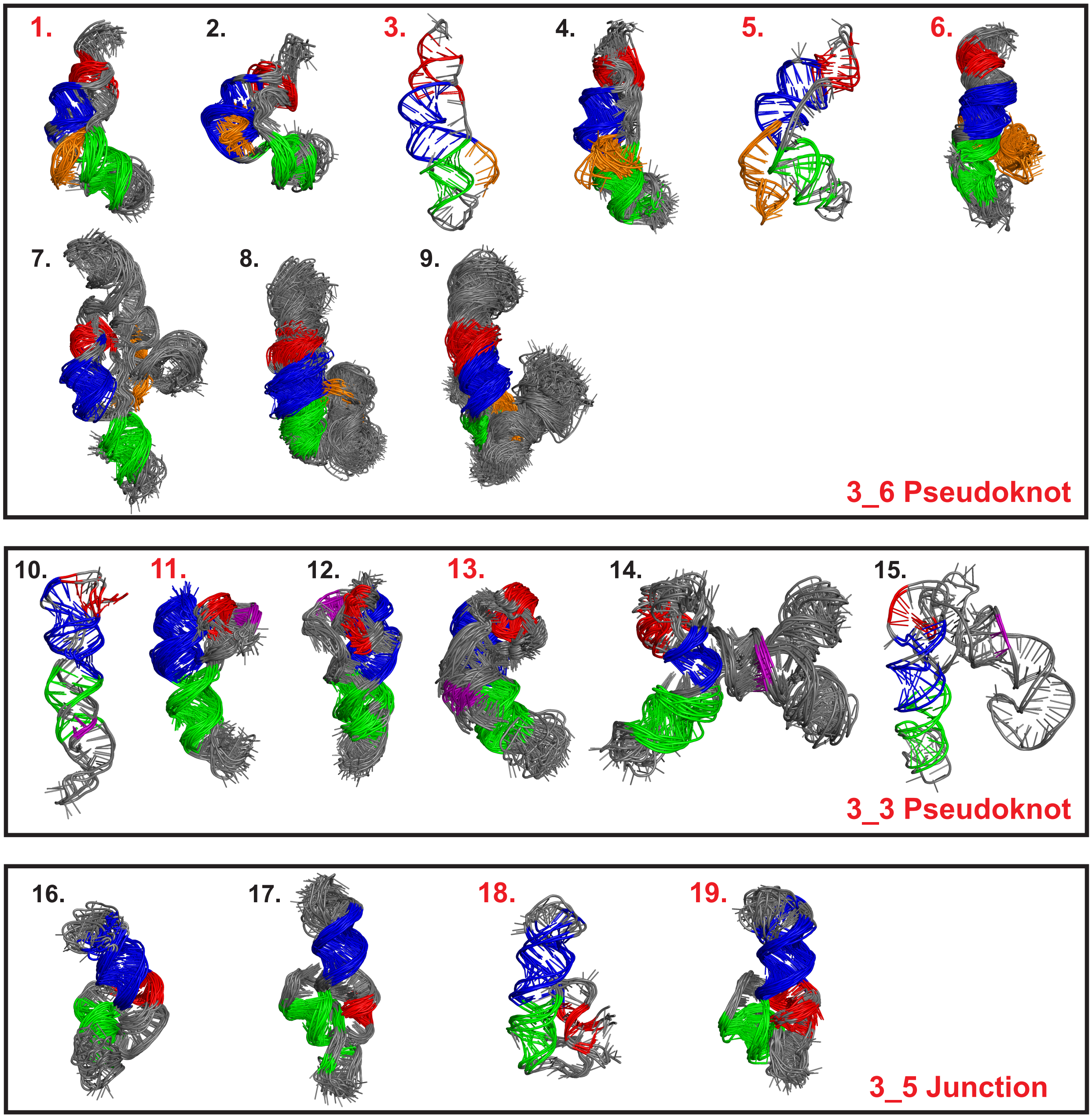

Supplementary Figure 8: Cluster center structures of the 19 validated wildtype systems, following enumeration in Table 1. Representative systems are numbered in red. For each trajectory, the centers of the top clusters that include 75% of the MD structures (Supplementary Fig. 7) are superimposed. Stem 1 is colored blue, Stem 2 red, and Stem 3 green. For 3_6, the 5′ end is colored orange. For 3_3, two 3′ end residues that form Stem 2 triplets in Fig. 6 are colored purple.

5 Mutant FSE models

Motif strengthening mutants in this work include 77-nt and 144-nt 3_6 pseudoknot, 77-nt 3_3 pseudoknot and 77-nt 3_5 junction. Mutants are predicted using the programs that have generated convergent and valid MD trajectories for the corresponding wildtype conformations. This leads to 11 predicted mutant systems (Supplementary Table 5).

Validation for mutant systems follows the same protocol as that for wildtype systems, including examination of graph topology and 2D structure for initial 3D predictions, and convergence for MD simulations. All the 11 initial predictions have correct motifs and consistent 2D structures with SHAPE.

Subsequent MD trajectories are examined for convergence and structural stability. All systems have steady NPT ensemble density (Supplementary Fig. 9). Simulations with large RMSD fluctuations were extended beyond 1 microsecond, including 77-nt 3_6 iFoldRNA, 77-nt 3_3 RNAComposer and iFoldRNA, and 144-nt 3_6 RNAComposer and iFoldRNA. All these systems reached stable RMSD plateau subsequently, except for 144-nt 3_6 RNAComposer (Supplementary Fig. 10). However, its RMSD fluctuations are caused by the flexible upstream and downstream stems, while its central 77-nt FSE region exhibits a relatively stable RMSD (shown as a dashed line in Supplementary Fig. 10). As its eRMSD, Rg, and H-bond evolutions are all stable (Supplementary Fig. 11, Supplementary Fig. 12, Supplementary Fig. 13), we regard these fluctuations inherent and do not exclude this system. All systems exhibit inherent and stable eRMSD (Supplementary Fig. 11), radius of gyration (Supplementary Fig. 12), and H-bond numbers (Supplementary Fig. 13).

Finally, all converged trajectories are tracked for 2D structure and motif. All trajectories maintain the correct motifs throughout the simulations, and their Stem 2 lengths are listed in Supplementary Table 5. Representative systems are: SimRNA for 77-nt 3_6, RNAComposer for 144-nt 3_6, iFoldRNA for 77-nt 3_3, and iFoldRNA for 77-nt 3_5.

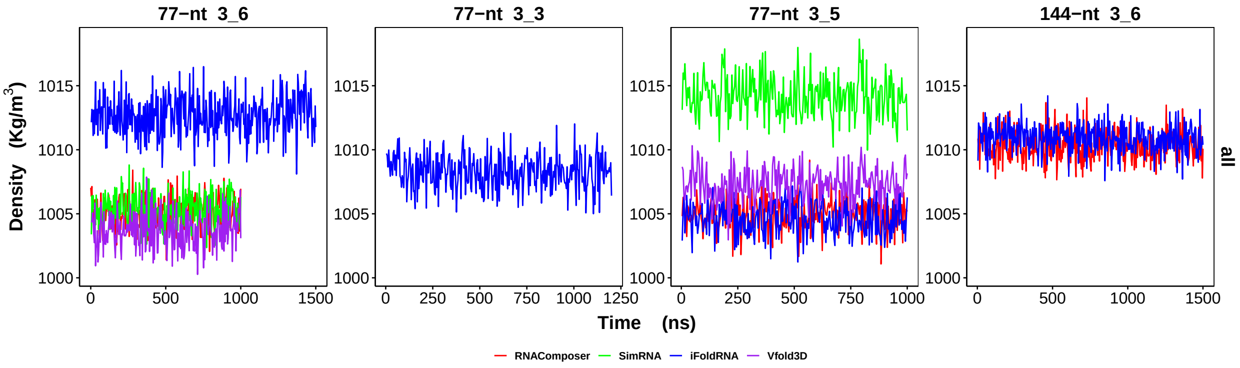

Supplementary Figure 9: Time evolution of system density in the 11 mutant FSE MD simulations.

Supplementary Table 5: Mutant FSE model validation. We monitor the Stem 2 length in the initial 3D model, MD start, MD middle, and MD end frames, as well as the largest cluster center structure. The trajectories are numbered following Table 1. Models with longest (i.e., more stable) Stem 2 are chosen as representatives and highlighted in yellow.

Conformer	Program		Stem 2 base pairs				
			Initial	MD start	MD mid	MD end	Cluster
77-nt M3_6	1′	RNAComp	9	9	4	4	4
	2′	SimRNA	9	9	8	9	9
	3’	iFoldRNA	8	9	6	7	7
	4’	Vfold3D	9	9	9	8	8
144-nt M3_6	7′	RNAComp	8	8	4	5	5
	8’	iFoldRNA	5	5	4	5	4
77-nt M3_3	11′	iFoldRNA	7	7	6	7	7
77-nt M3_5	16′	RNAComp	6	7	7	6	7
	17′	SimRNA	6	7	7	7	7
	18′	iFoldRNA	6	7	7	7	7
	19′	Vfold3D	7	6	6	6	6

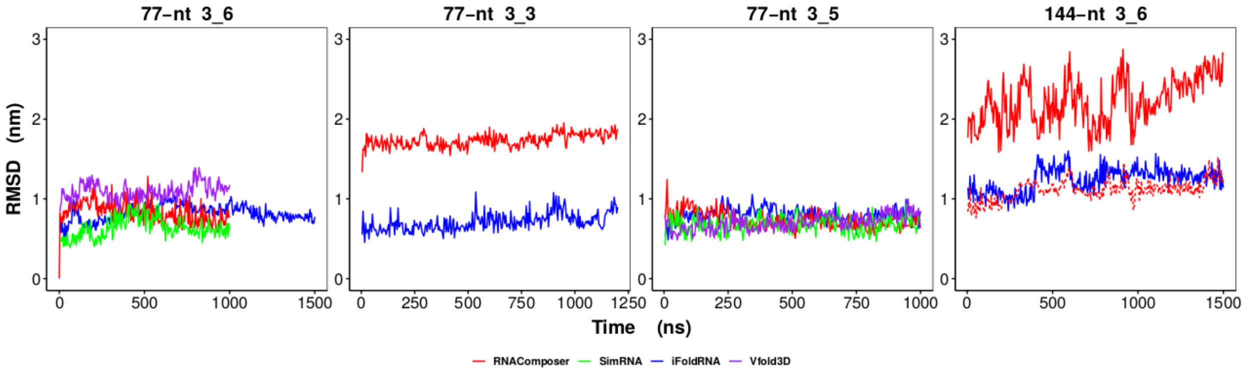

Supplementary Figure 10: Time evolution of RMSD in the 11 mutant FSE MD simulations. For the 144-nt 3_6 RNAComposer (red) mutant system, we also plot the RMSD evolution for the central 77-nt FSE region (dashed).

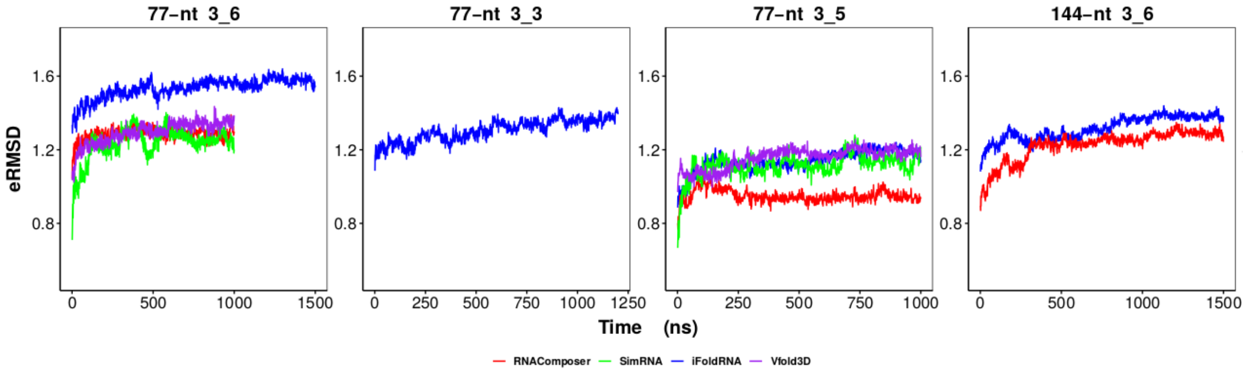

Supplementary Figure 11: Time evolution of eRMSD in the 11 mutant FSE MD simulations.

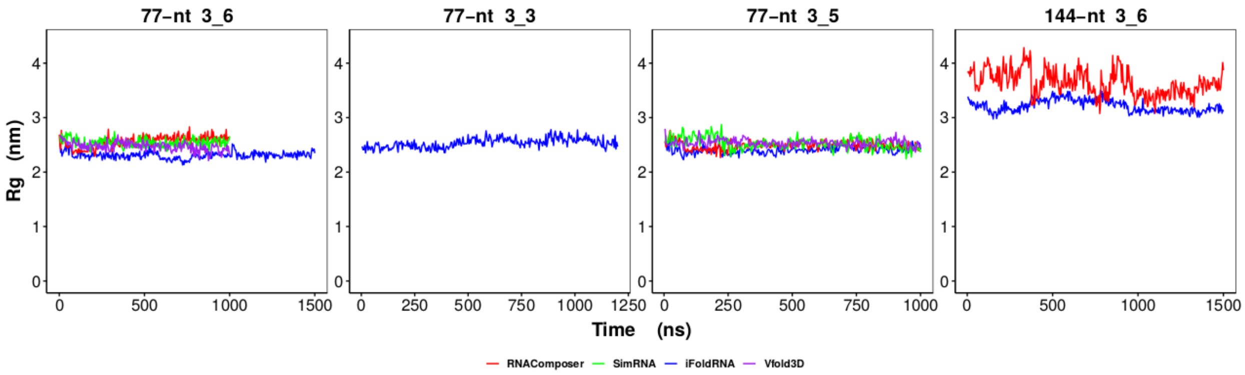

Supplementary Figure 12: Time evolution of the radius of gyration in the 11 mutant FSE MD simulations.

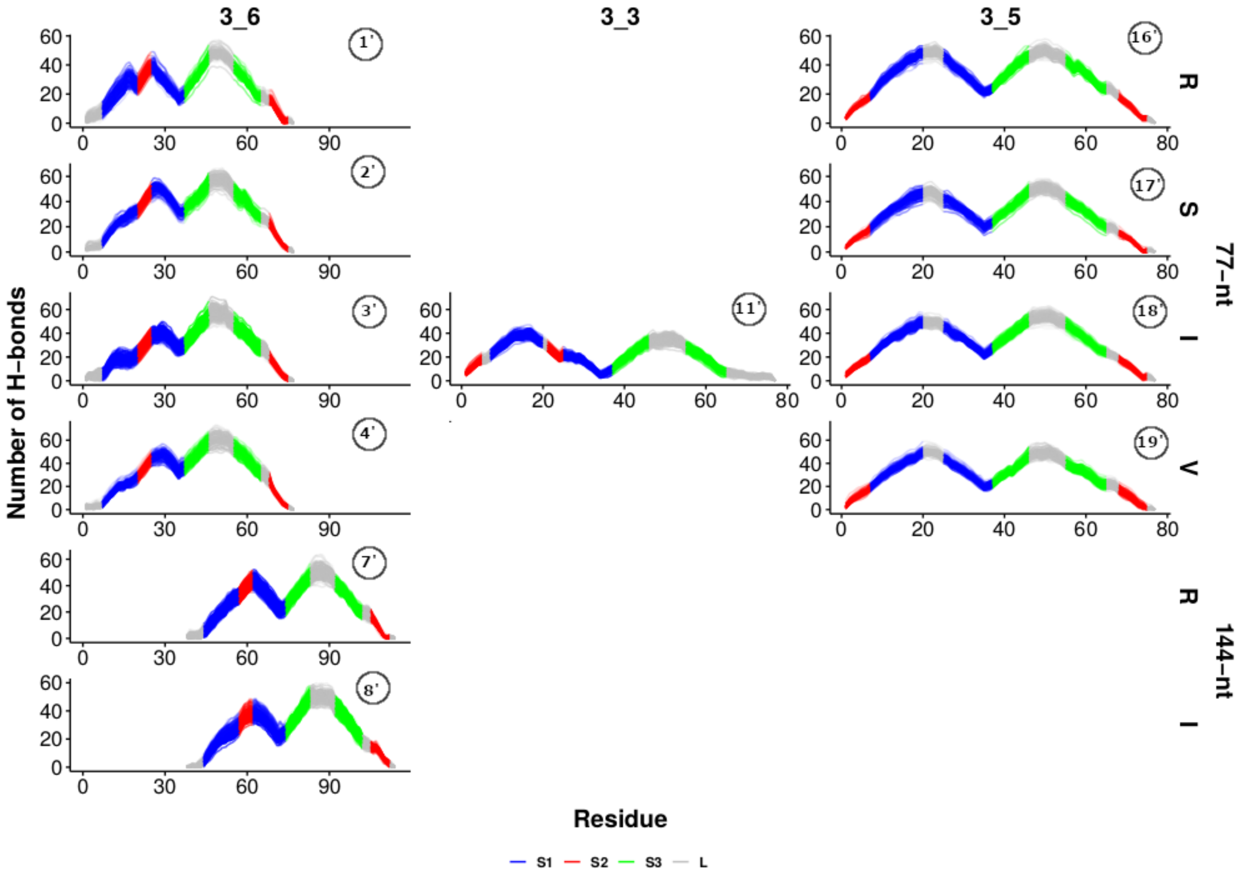

Supplementary Figure 13: Hydrogen bonds in the 11 mutant FSE MD simulations, with trajectory number as defined in Table 1. The cumulative number of hydrogen bonds by residue is calculated, with S1 residues in blue, S2 in red and S3 in green. Structures for analysis are extracted every 100 ns in MD simulations.

Supplementary Table 6: Comparison of the motif-strengthening mutants to the wildtype systems. The trajectories are numbered following Table 1. Representative systems are highlighted in yellow. For 3_6, we check whether there is change in the 5′ end threading and the structure shape. For 3_3, we search for the Stem 2 triplets seen in Fig. 6 and the flanking stem SF. For 3_5, we check for co-axial stacking and the shape.

Trajectory	Mutant		Wildtype	
**3_6 Pseudoknot**	**Threaded**	**Shape**	**Threaded**	**Shape**
1′ 77-nt R	Yes	Linear	Yes	L
2′ 77-nt S	Yes	Linear	Yes	L
3′ 77-nt I	No	Linear	No	Linear
4′ 77-nt V	No	Linear	Yes	Linear
7′ 144-nt R	No	Linear	No	Linear
8′ 144-nt I	No	Linear	No	Linear
**3_3 Pseudoknot**	**Stem 2 triplets**	**Stem SF**	**Stem 2 triplets**	**Stem SF**
11′ 77-nt I	No	No	Yes	No
**3_5 Junction**	**Co-axial stacking**	**Shape**	**co-axial stacking**	**Shape**
16′ 77-nt R	S1, S2	Elongated	S1, S2	Compact
17′ 77-nt S	S1, S2	T shape	S1, S2	Elongated
18′ 77-nt I	S1, S2	Elongated	S1, S3	Compact
19′ 77-nt V	S1, S2	Elongated	S1, S2	Elongated

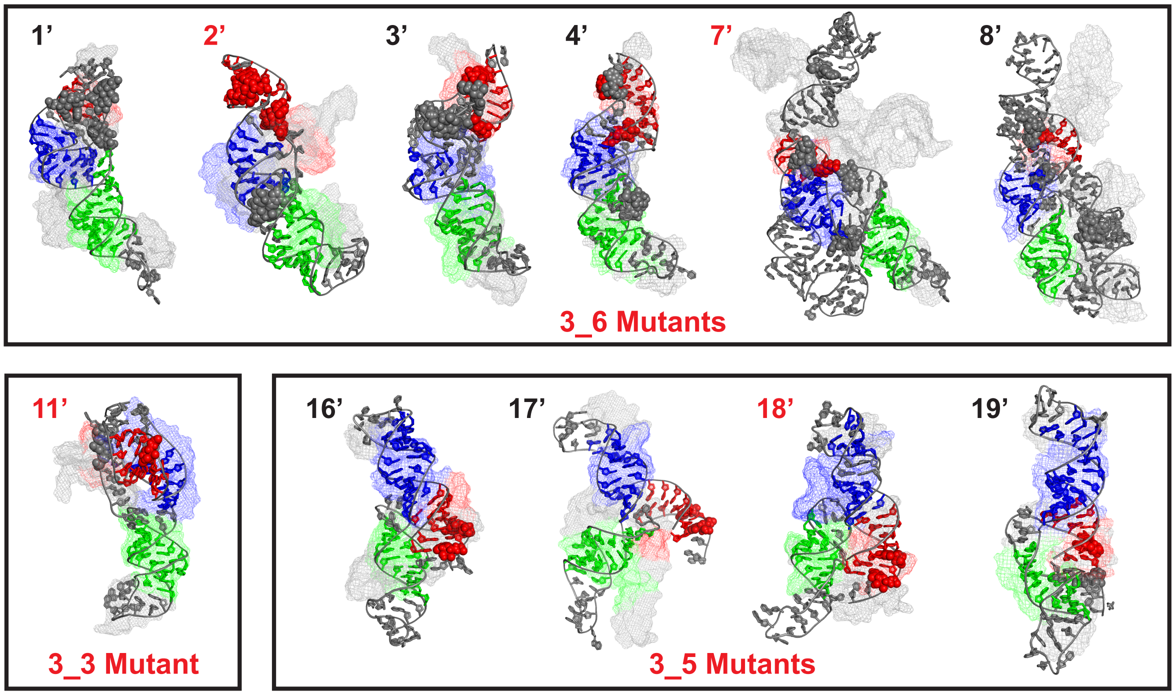

Supplementary Figure 14: Comparison of the motif-strengthening mutants to the wildtype systems. The largest cluster center structures of the mutants (shown in cartoon mode) are aligned with those of the wildtype (mesh mode), following enumeration in Table 1. Representative systems are numbered in red. Stem 1 is colored blue, Stem 2 red, and Stem 3 green. The mutated residues are drawn as spheres by PyMol.

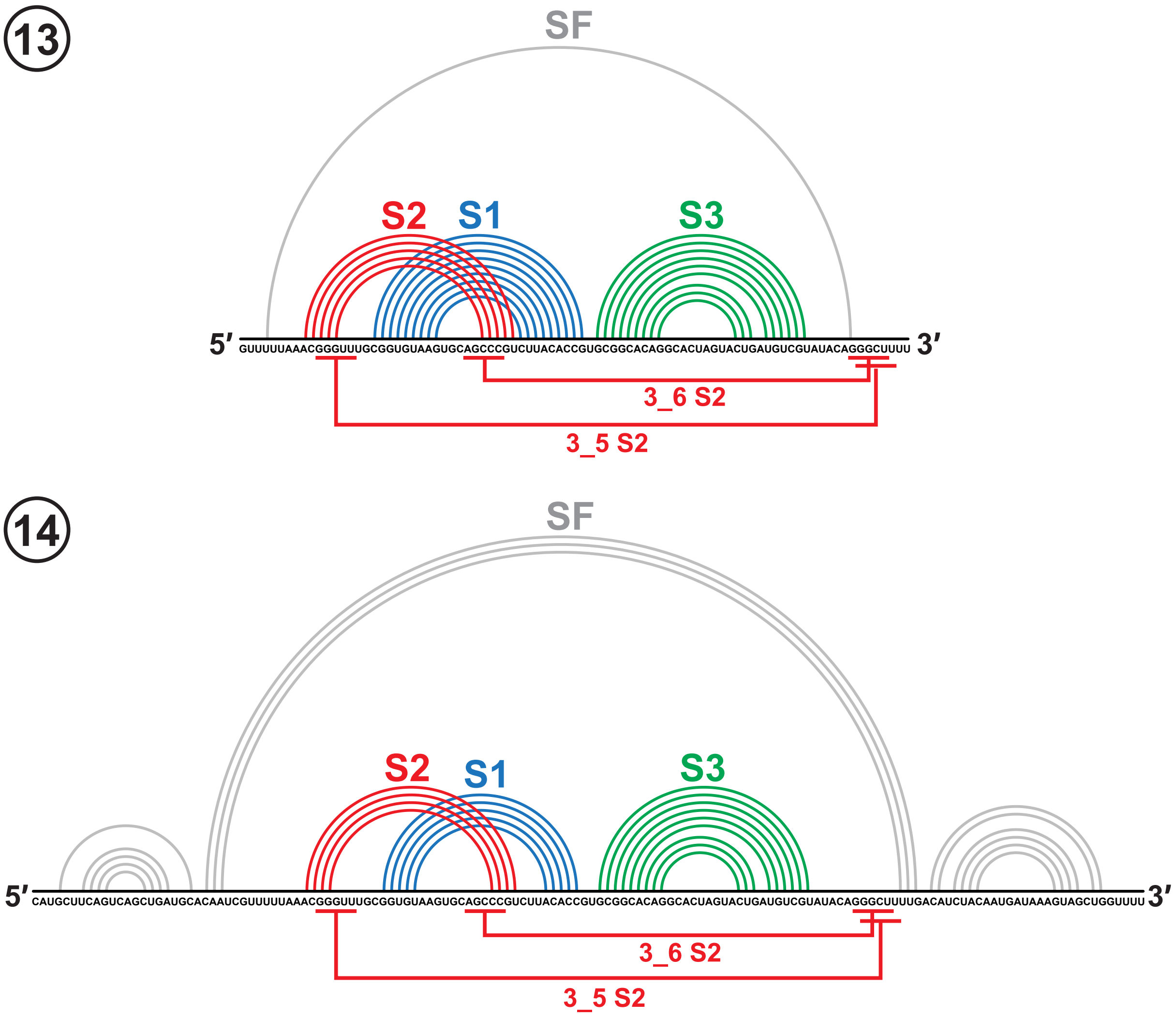

Supplementary Figure 15: Secondary structures of the 3_3 pseudoknot for 87 and 144-nt FSE models are shown as arc plots at top, with trajectories labeled as in Table 1. The 3 stems and the flanking stem SF are labeled. The alternative Stem 2 of 3_6 and 3_5 are indicated at bottom. As we can see, formation of stem SF blocks these alternative Stem 2.

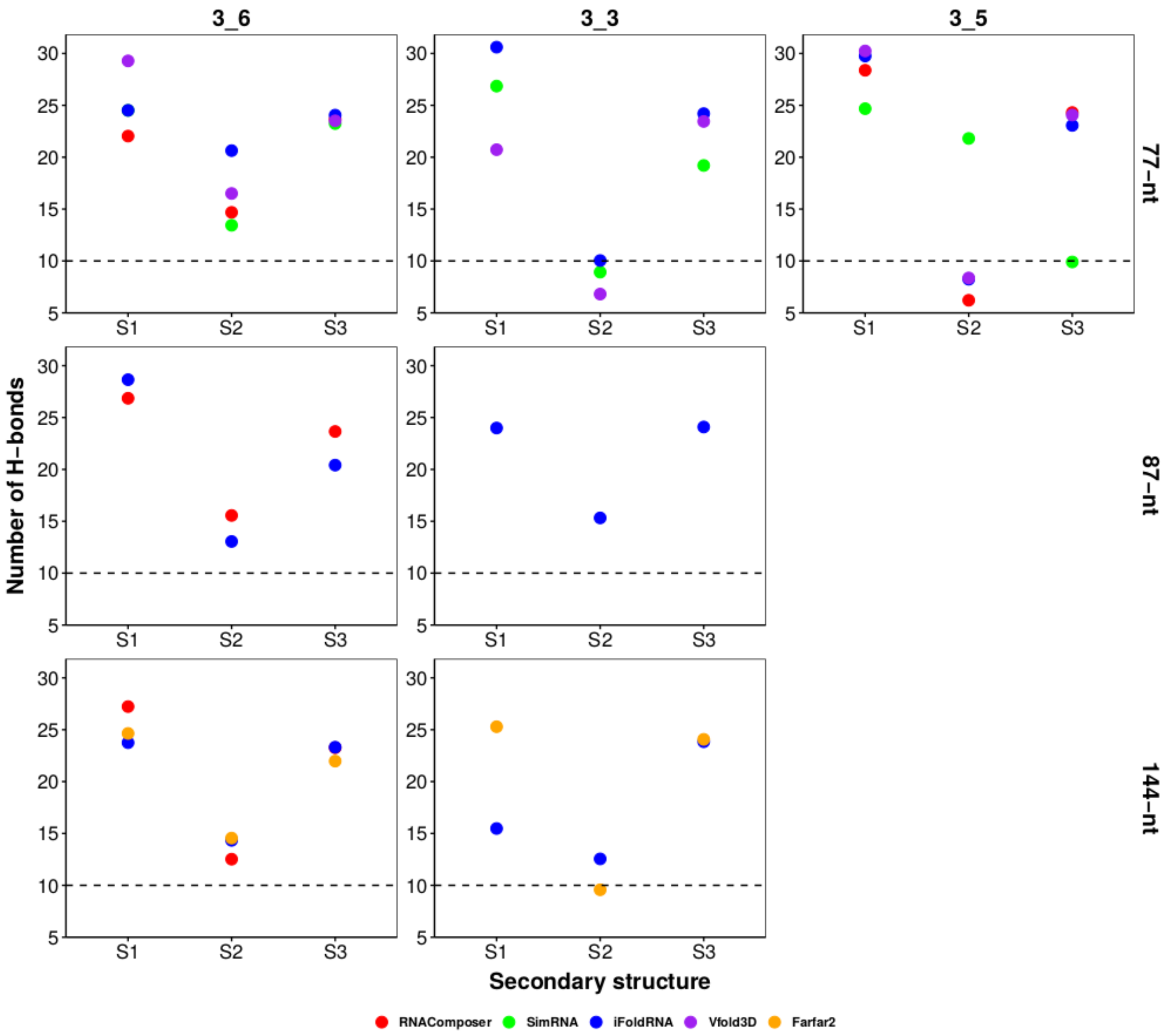

Supplementary Figure 16: Number of H-bonds in the three stems of the wildtype 3_6, 3_3, and 3_5 conformations for 77, 87, and 144-nt. Stem 2 is weakest in all three motifs. At 77-nt, 3_6 has the strongest Stem 2. At 87-nt, 3_3 has a slightly stronger Stem 2 than 3_6.

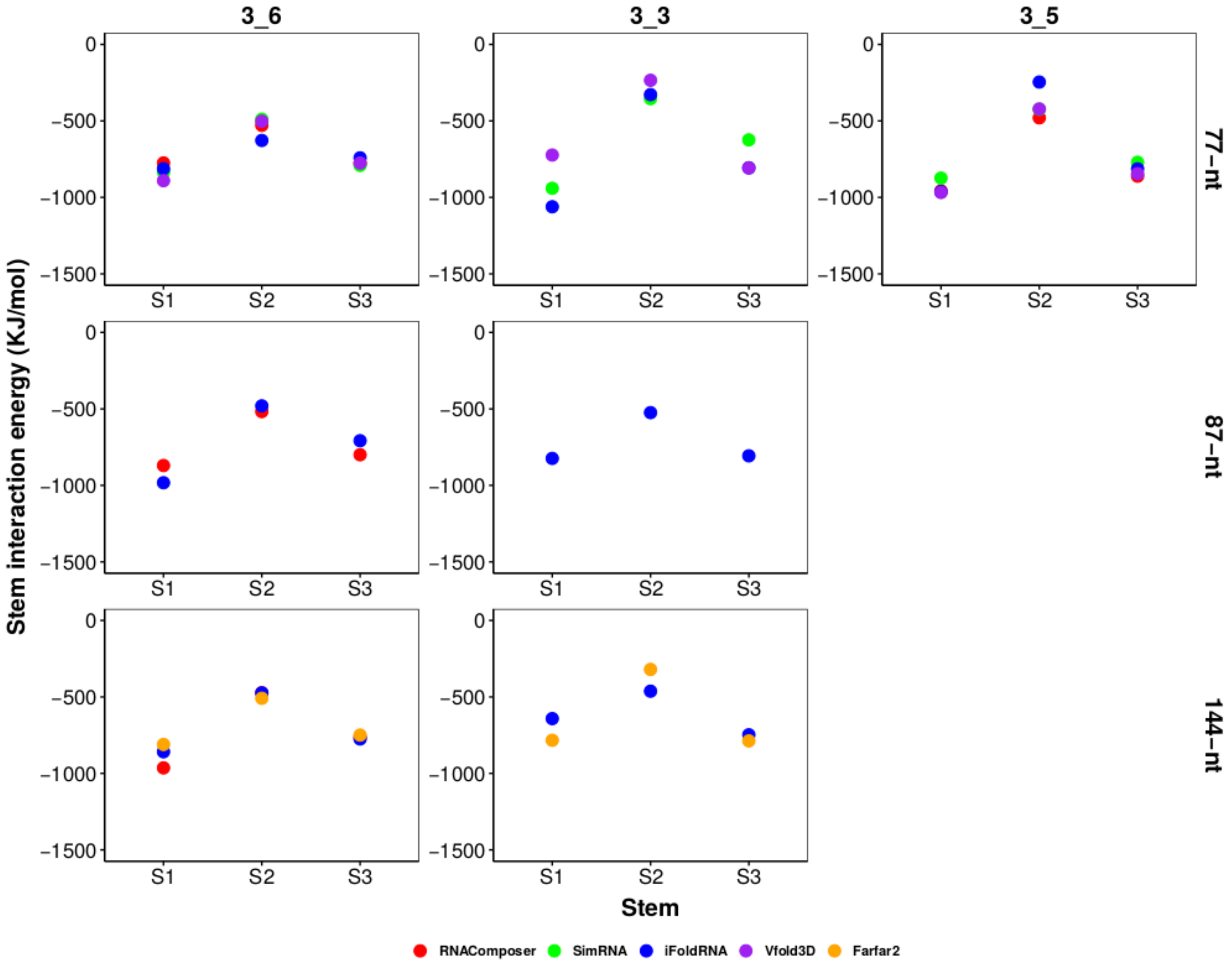

Supplementary Figure 17: Interaction energy between the strands in the three stems of the wildtype 3_6 and 3_3 conformations for 77, 87, and 144-nt.

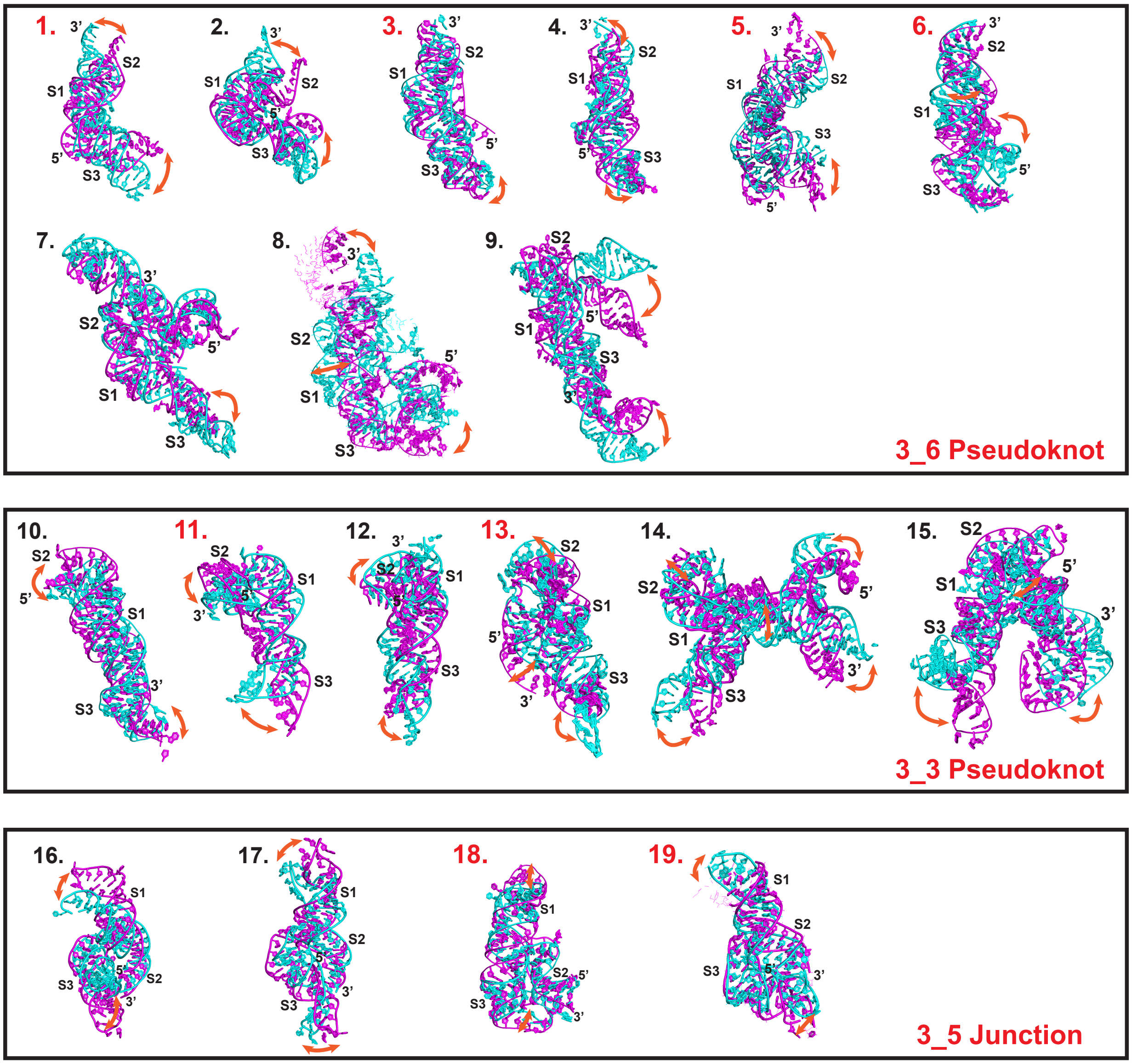

Supplementary Figure 18: Dominant motions in the 19 validated wildtype systems revealed by principal component analysis, with trajectory number as defined in Table 1. Representative systems are numbered in red. Two extreme frames are extracted and colored in magenta and cyan. The stems and the 5′ and 3′ ends are labeled when visible, and the motions are highlighted using arrows. In trajectories 8 and 19, some of residue distances in the two extreme frames are higher than usual, and these residues can only be visualized using line drawing method in PyMol.

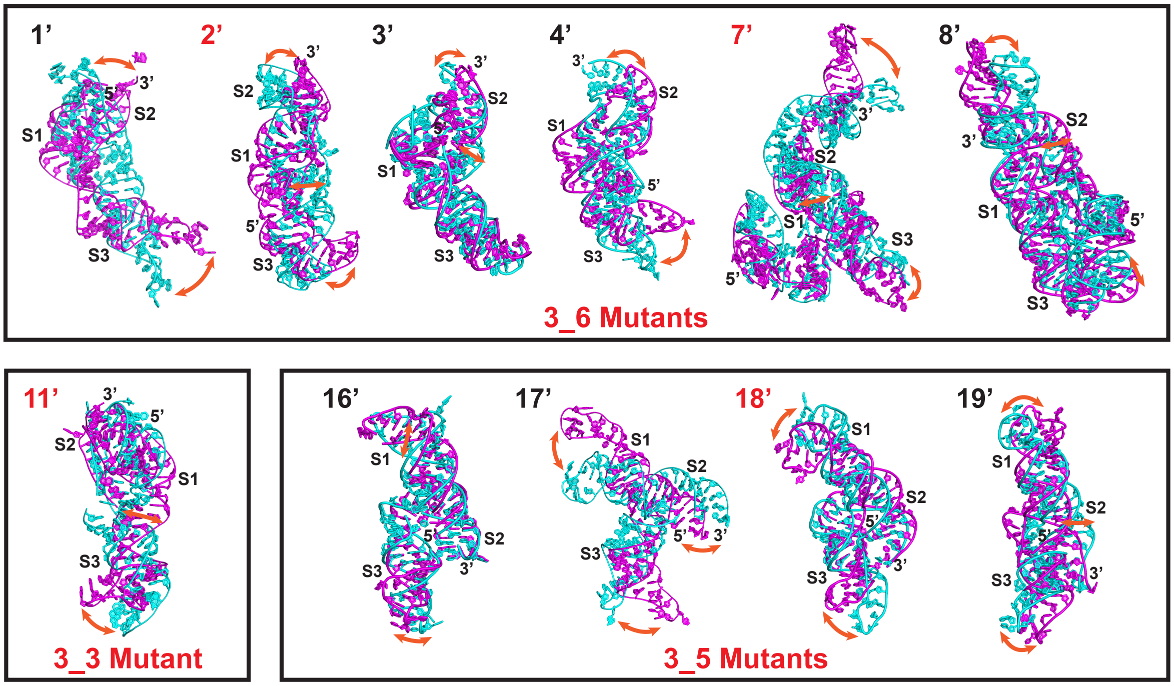

Supplementary Figure 19: Dominant motions in the 11 validated mutant systems revealed by principal component analysis, with trajectory number as defined in Table 1. Representative systems are numbered in red. Two extreme frames are extracted and colored in magenta and cyan. The stems and the 5′ and 3′ ends are labeled when visible, and the motions are highlighted using arrows.

Supplementary references

(1) Elbe, S.; Buckland-Merrett, G. Data, disease and diplomacy: GISAID's innovative contribution to global health. *Glob. Chall.* **2017**, *1*, 33–46.

(2) Schlick, T.; Zhu, Q.; Dey, A.; Jain, S.; Yan, S.; Laederach, A. To Knot or Not to Knot: Multiple Conformations of the SARS-CoV-2 Frameshifting RNA Element. *J. Amer. Chem. Soc.* **2021**, *143*, 11404–11422.

(3) Biesiada, M.; Purzycka, K.; Szachniuk, M.; Blazewicz, J.; Adamiak, R. Automated RNA 3D Structure Prediction with RNAComposer. *Methods Mol. Biol.* **2016**, *1490*, 199–215.

(4) Boniecki, M.; Lach, G.; Dawson, W.; Tomala, K.; Lukasz, P.; Soltysinski, T.; Rother, K.; Bujnicki, J. SimRNA: a coarse-grained method for RNA folding simulations and 3D structure prediction. *Nucleic Acids Res.* **2016**, *44*, e63–e63.

(5) Krokhotin, A.; Houlihan, K.; Dokholyan, N. iFoldRNA v2: folding RNA with constraints. *Bioinformatics* **2015**, *31*, 2891–2893.

(6) Xu, X.; Chen, S. Hierarchical Assembly of RNA Three-Dimensional Structures Based on Loop Templates. *J. Phys. Chem. B* **2018**, *122*, 5327–5335.

(7) Watkins, A.; Rangan, R.; Das, R. FARFAR2: Improved De Novo Rosetta Prediction of Complex Global RNA Folds. *Structure* **2020**, *28*, 963–976.e6.

(8) Lu, X.; Bussemaker, H.; Olson, W. DSSR: an integrated software tool for dissecting the spatial structure of RNA. *Nucleic Acids Res.* **2015**, *43*, e142–e142.

(9) Bottaro, S.; Bussi, G.; Pinamonti, G.; Reißer, S.; Boomsma, W.; Lindorff-Larsen, K. Barnaba: software for analysis of nucleic acid structures and trajectories. *RNA* **2019**, *25*, 219–231.

(10) Bottaro, S.; Di Palma, F.; Bussi, G. The role of nucleobase interactions in RNA structure and dynamics. *Nucleic Acids Res.* **2014**, *42*, 13306–13314.

(11) Williams, C.; Headd, J.; Moriarty, N.; Prisant, M., et al. MolProbity: More and better reference data for improved all-atom structure validation. *Protein Sci.* **2018**, *27*, 293–315.

(12) Jones, C.; Ferre-D'amare, A. Crystal structure of the severe acute respiratory syndrome coronavirus 2 (SARS-CoV-2) frameshifting pseudoknot. *RNA* **2022**, *28*, 239–249.

(13) Roman, C.; Lewicka, A.; Koirala, D.; Li, N.; Piccirilli, J. The SARS-CoV-2 Programmed −1 Ribosomal Frameshifting Element Crystal Structure Solved to 2.09 Å Using Chaperone-Assisted RNA Crystallography. *ACS Chem. Biol.* **2021**, *16*, 1469–1481.

(14) Bhatt, P.; Scaiola, A.; Loughran, G.; Leibundgut, M.; Kratzel, A., et al. Structural basis of ribosomal frameshifting during translation of the SARS-CoV-2 RNA genome. *Science* **2021**, *372*, 1306–1313.

(15) Zhang, K.; Zheludev, I.; Hagey, R.; Haslecker, R., et al. Cryo-EM and antisense targeting of the 28-kDa frameshift stimulation element from the SARS-CoV-2 RNA genome. *Nat. Struct. Mol. Biol.* **2021**, *28*, 747–754.

(16) Schrödinger, LLC, The PyMOL Molecular Graphics System, Version 1.8. **2015**,
